# Framework Dimensional Control Boosting Charge Storage in Conjugated Coordination Polymers

**DOI:** 10.1002/advs.202205760

**Published:** 2022-12-09

**Authors:** Kun Fan, Cheng Fu, Yuan Chen, Chenyang Zhang, Guoqun Zhang, Linnan Guan, Minglei Mao, Jing Ma, Wenping Hu, Chengliang Wang

**Affiliations:** ^1^ School of Optical and Electronic Information Wuhan National Laboratory for Optoelectronics (WNLO) Huazhong University of Science and Technology Wuhan 430074 China; ^2^ Wenzhou Advanced Manufacturing Technology Research Institute Huazhong University of Science and Technology Wenzhou 325035 China; ^3^ School of Chemistry and Chemical Engineering Nanjing University Nanjing 210093 China; ^4^ Tianjin Key Laboratory of Molecular Optoelectronic Sciences Department of Chemistry School of Sciences Tianjin University Tianjin 300072 China

**Keywords:** cathodes, conductive metal‐organic frameworks, conjugated coordination polymers, dual‐ion batteries, organic sodium‐ion batteries

## Abstract

Conjugated coordination polymers (CCPs) with extended *π*–d conjugation, which can effectively promote long‐range delocalization of electrons and enhance conductivity, are superior to traditional metal‐organic frameworks (MOFs) and attracted great attention for potential applications in chemical sensors, electronics, energy conversion/storage devices, etc. However, the precise construction of CCPs is still challenging due to the complex and uncontrollable reactions of CCPs. Herein, two different framework dimensions of CCPs are controllably realized by employing the same ligand (2,3,5,6‐tetraaminobenzoquinone (TABQ)) and the same metal (copper) as center ions. The manipulation of reaction leads to different valences of ligands and metal ions, different coordination geometries, and thereby 1D‐CuTABQ and 2D‐CuTABQ frameworks, respectively. High performance of charge storage is hence achieved involving the storage of both cations and anions, and therein, 2D‐CuTABQ shows a high reversible capacity of ≈305 mAh g^−1^, good rate capability and high capacity retention (≈170 mAh g^−1^ after 2000 cycles at 5 A g^−1^ with 0.01% decay per cycle), which outperforms 1D‐CuTABQ and almost all of the reported MOFs as cathodes for batteries. These results highlight the delicate structural control of CCPs for high‐performance batteries and other various applications.

## Graphical abstract

The framework dimensions of conjugated coordination polymers (CCPs) are controllably realized by employing 2,3,5,6‐tetraaminobenzoquinone (TABQ) as ligands and copper ions as center ions. High performance of charge storage is hence achieved involving the storage of both cations and anions, showing performance superior to the reported metal‐organic frameworks (MOFs) as cathodes for batteries.

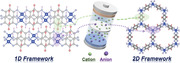

## 1 Introduction

The emerging conjugated coordination polymers (CCPs) as a unique kind of metal‐organic frameworks (MOFs) have the extra advantages of high electrical conductivity and stability compared with traditional MOFs, in virtue of *π*–d hybridization between the conjugated ligands and transition metal ions, enabling the long‐range delocalization of electrons on the whole skeleton and hence have been widely investigated for diverse applications,^[^ ^1^ ^]^ ranging from semiconductors,^[^ ^2^ ^]^ superconductors,^[^ ^3^ ^]^ sensors^[^ ^4^ ^]^ to electrocatalysis,^[^ ^5^ ^]^ energy storage devices,^[^ ^6^ ^]^ and so on.^[^ ^7^ ^]^

**Figure 1:**
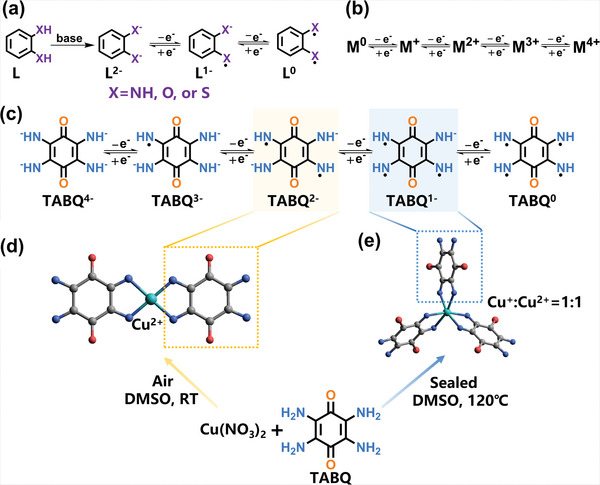
a) Possible chemical states of ligand fragment and b) different valance states of metal ions. c) Possible redox states of the linker TABQ without accounting the redox of carbonyl groups for better understanding. Noted that in principle, TABQ could accept one or two more electrons due to the reduction of carbonyl groups that might probably occur between the above diagramatic −2 and −3 and will be discussed below. d) Illustration of the square planar building unit in one‐dimensional frameworks and the synthesis route. e) Illustration of the octahedral building unit in two‐dimensional frameworks and the synthesis route.

However, both the conjugated ligand and the metal center may suffer in situ oxidation/reduction during the synthesis (**Figure** **1**a,b).^[^ ^8^ ^]^ The simultaneous deprotonation of ligands, oxidation/reduction of both ligands and metal ions and coordination between them always result in the complex and uncontrollable reactions, multifarious chemical states and frameworks and low crystallinity of CCPs.^[^ ^9^ ^]^ For example, the copper centers could display either +2, +1, or even 0 valence during the synthesis.^[^ ^8^, ^10^ ^]^ On the other hand, the bidentate function group in the ligands could form either −2, −1, or even 0 valence, leading to a variety of valence state of the conjugated ligand at certain circumstance.^[^ ^9^, ^11^ ^]^ An example ligand, 2,3,5,6‐tetraaminobenzoquinone (TABQ) with different chemical states is shown in Figure 1c.^[^ ^12^ ^]^ Hence, the precise construction of CCPs is still challenging.^[^ ^12^, ^13^ ^]^

**Figure 2:**
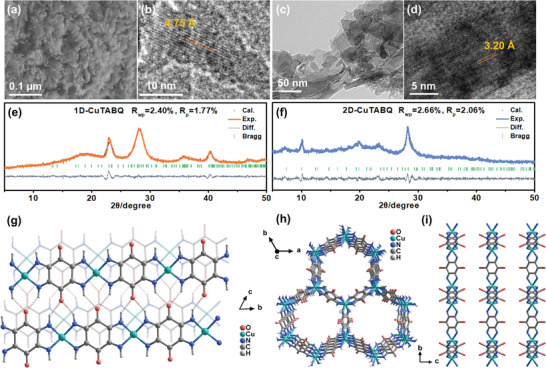
a) SEM and b) HRTEM images of 1D‐CuTABQ. c,d) TEM and HRTEM images of 2D‐CuTABQ, respectively. Rietveld refinement of PXRD patterns of e) 1D‐CuTABQ and f) 2D‐CuTABQ. g) The slipped *π*‐stacking chain structure of 1D‐CuTABQ, the chains were further interconnected by hydrogen bonding between −NH and C = O (dashed lines). h,i) Structures of two‐dimensional layer of 2D‐CuTABQ, viewed along the crystallographic *c* axis and *a* axis, respectively.

Herein, different framework dimensions of CCPs were controllably realized by employing TABQ as ligands and copper ions as center ions. The manipulation of reaction led to the different valences of ligands and metal ions, different coordination geometries, and thereby 1D‐CuTABQ (Figure 1d) and 2D‐CuTABQ (Figure 1e) frameworks, respectively. High performance of charge storage was hence achieved involving the storage of both cations and anions, which is though challenging, but quite attractive for high capacity, high output voltage and thereby high energy and power density for batteries.^[^ ^14^ ^]^ Benefiting from the unique 2D framework, the 2D‐CuTABQ showed great potential in charge storage, involving the reduction of ligand and pristine cupric (Cu^2+^) ions that accompanied with intercalation/deintercalation of Na^+^ and the oxidation of pristine cuprous (Cu^+^) ions that charge‐balanced by the storage of PF_6_^−^ anions. As a result, the 2D‐CuTABQ cathode delivered a high reversible capacity of ≈305 mAh g^−1^ at 0.1 A g^−1^ and a high rate performance along with remarkable cycling stability (≈170 mAh g^−1^ after 2000 cycles at 5 A g^−1^ with 0.01% decay per cycle), which are superior to 1D‐CuTABQ and almost all of the previously reported MOFs as cathodes for batteries. These results highlight the delicate structural control of CCPs for high‐performance batteries and other various applications.

## 2 Results and Discussion

### 2.1 Synthesis and Characterization

1D conjugated coordination polymers of Cu‐TABQ was synthesized according to our previous work on the Ni‐BTA (where BTA = 2,3,5,6‐tetraaminobenzene).^[^ ^6d^ ^]^ Our previous work has shown that the slow reaction process will benefit the precise coordination of nitrogen atoms with metal ions within a square‐planar configuration (see details in the Supporting Information). An air atmosphere and a base are required for the in situ deprotonation and oxidation of the ligands.^[^ ^8^, ^10^ ^]^ The as‐obtained 1D‐CuTABQ exhibited rod‐like morphology, although the rod was quite short, ranging from a few nanometers to 100 nm as shown in the scanning electron microscopy (SEM) and transmission electron microscopy (TEM) images (**Figure** **2**a and Figure S1, Supporting Information). On the other hand, previous works have shown that high temperature may result in the different coordination configurations of CCPs.^[^ ^15^ ^]^ Hence, the 2D‐CuTABQ was achieved by solvothermal reactions of Cu(NO_3_)_2_ with TABQ in DMSO at 120 °C for 72 h, which afforded a black powder with sheet morphology (Figure 2c and Figure S2, Supporting Information). Different from the synthesis of 1D‐CuTABQ, base and extra oxygen are absent in the sealed tube, which may lead to the different valence of both ligand and center metal ions that will be further discussed in the following.

**Figure 3:**
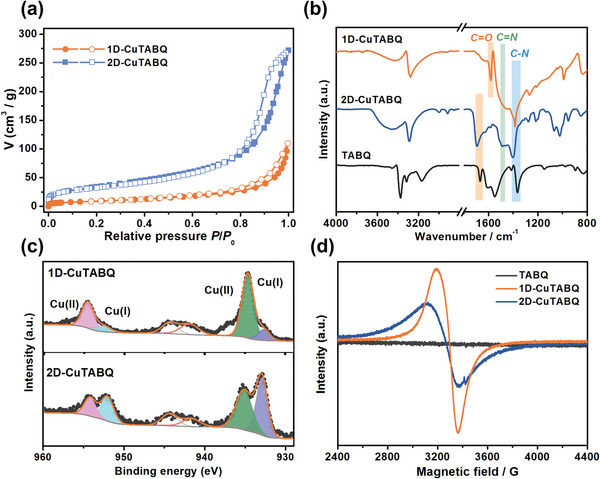
a) N_2_ adsorption (filled circle) and desorption (open circle) isotherms of 1D‐CuTABQ and 2D‐CuTABQ, respectively. b) FTIR spectra of the free ligand TABQ, 1D‐CuTABQ, and 2D‐CuTABQ, c) Cu 2p XPS spectra and d) EPR spectra of the 1D‐CuTABQ and 2D‐CuTABQ.

To confirm the formation of CCPs with different framework dimensions, diverse characterizations were carried out. The elemental analysis results showed that the molar ratio of Cu:TABQ was 1:1 and 1:1.5, respectively (Table S1, Supporting Information). No other counter ions were observed. Such results strongly indicated that the two products may have different coordination configurations. The coordination geometry of 1D‐CuTABQ should be considered to be a square planar, forming a 1D chain structure, similar to that of Ni‐BTA,^[^ ^6d^ ^]^ 1D‐Ni‐TABQ^[^ ^12^, ^16^ ^]^ and other ideal 1D CCPs. On the other hand, the secondary building units (SBUs) in 2D‐CuTABQ should be present as tris‐chelating clusters, similar to the SBUs in the 2D structure based on chloranilate (Cl_2_dhbq) ligands.^[^ ^17^ ^]^ The N_2_ sorption isotherms showed that the 2D‐CuTABQ had larger BET surface area of ≈124.5 m^2^ g^−1^ and pore volume of 0.42 cm^3^ g^−1^ than those of 1D‐CuTABQ (34.3 m^2^ g^−1^ and 0.17 cm^3^ g^−1^), confirming the difference in framework dimensions between the two products (**Figure** **3**a). TGA curves showed good thermal stability for both two samples (Figure S3, Supporting Information). Moreover, the weight loss in the thermal analyses is coincident well with the proposed molecular formula Cu(C_6_H_4_N_4_O_2_)(DMSO)_0.025_(H_2_O) for 1D‐CuTABQ and Cu(C_6_H_4_N_4_O_2_)_1.5_(DMSO)_0.5_(H_2_O) for 2D‐CuTABQ based on elemental analysis (Table S1, Supporting Information). Both the energy dispersive X‐ray spectrum (EDS) mappings of the 1D‐CuTABQ and 2D‐CuTABQ indicated that the elements were evenly distributed in the material (Figures S4 and S5, Supporting Information). As shown in FT‐IR spectra (Figure 3b), the characteristic vibrations of –NH_2_ in TABQ at around 3000–3500 cm^−1^ disappeared in both products, and a clear signal of –NH– stretching vibration at 3290 cm^−1^ appeared, indicating the deprotonation of amino groups and formation of coordination bonds between –NH– atoms and metal cations.^[^ ^16^ ^]^ The stretching vibration of carbonyl bond (C=O) in 1D‐CuTABQ underwent a red shift from 1670 to 1581 cm^−1^ owing to the efficient electron delocalization in a conjugated system, which was similar to the related materials containing the dianionic TABQ^2−^ ligand.^[^ ^12^, ^16^ ^]^ However, the C=O absorption in 2D‐CuTABQ shifted to high frequency (1693 cm^−1^) compared with the free ligand, which is quite different from the typical bathochromic shift in the other CCPs that involved coordination of O atoms with metal node.^[^ ^18^ ^]^ This result demonstrated that only the N atoms were coordinated with Cu ions and the C=O groups hung from the skeleton as free groups. The broad peak at 1485 cm^−1^ of both materials could be assigned to the C=N bonds. While the peaks at 1400 cm^−1^ could be assigned to C—N bonds. The coexistence of double (C=N) and single (C—N) bond characters could also be confirmed by N 1s spectra in X‐ray photoelectron spectroscopy (XPS) (Figures S6 and S7, Supporting Information). These results indicated that although the two materials had different coordination configurations, both of them had similar coordination bonds (Cu—(NH)_4_) and the C=O groups did not participate in the coordination. In addition, the high‐resolution XPS of Cu (2p) spectra revealed that most of the Cu ions are Cu^2+^ species (≈5/6) in 1D‐CuTABQ; while the ratio of Cu^2+^ and Cu^+^ species in 2D‐CuTABQ was about 1:1 (Figure 3c). Under air atmosphere and room temperature, the four amino groups in TABQ underwent an in situ deprotonation (forming TABQ^4−^) and oxidation process, and then the formed dianionic TABQ^2−^ ligand *bi*‐chelated with square‐planar Cu ions (dominant as +2 valence) to form a 1D chain framework (1D‐CuTABQ). Thereinto, adequate oxygen from air acted as the oxidant and hence most of the Cu ions kept as Cu^2+^ species. The valences of ligands and metal ions are −2 and +2, respectively, resulting in the molar ratio of Cu:TABQ of 1:1 as mentioned above. However, in a sealed tube at 120 °C, the TABQ ligands could be further oxidized to valence of −1 by either Cu^2+^ ions or the residual oxygen in the tube. Hence, more Cu ions were reduced to the valence of +1, due to insufficient oxygen in the confined space. Such mixed valences of copper have been previously observed in copper based MOFs, in which the redox‐active ligand was the reductant during the syntheses.^[^ ^8^, ^10^, ^19^ ^]^ The valences of TABQ ligand could be inferred from the molar ratio of Cu:TABQ (1:1.5) and the average valence of Cu ions (+1.5). The variation of valences of both ligand and metal ions and the adaptation of coordination geometry led to a unique neutral 2D framework (Figure 1). The variable valence states of TABQ ligands have been confirmed in other similar structures.^[^ ^12^ ^]^ Electron paramagnetic resonance (EPR) spectrum of 1D‐CuTABQ indeed showed a broad EPR signal with a *g*‐factor of 2.103, which was attributed to the existence of Cu(II). While for 2D‐CuTABQ, apart from this broad EPR signal, another EPR signal at *g* = 2.005 (Figure 3d) could be identified as the ligand radicals, which is consistent with the previously reported CCPs.^[^ ^8^, ^20^ ^]^ Theoretically, radicals should be also observed in 1D‐CuTABQ (also see the following), although the reason is not clear (probably similar with the dihydroxybenzoquinonate (dhbq)‐based CCPs,^[^ ^18a^ ^]^ or due to the more prominent Cu(II) leading to the relatively strong signal of Cu(II) and the overlap with the radical signals). Both the valence changes in ligand and metal ions indicated that both compounds should have excellent redox activity, which can be used as electrodes for batteries.^[^ ^6^, ^21^ ^]^ The redox‐active metal ions and organic ligands will result in multiple electrons transfer and hence high capacity.

To better understand the coordination configuration and framework structures, the skeletons and molecular arrangement of the two materials were optimized by density functional theory (DFT) calculations and further refined by powder X‐ray diffraction (PXRD) data. As shown in Figure 2e, the experimental and simulated results of 1D‐CuTABQ matched well, with low *R*_p_ of 1.77% and *R*_wp_ of 2.40%.The 1D‐CuTABQ crystallized in the triclinic space group with cell parameters of *a* = 6.3792 Å, *b* = 7.7279 Å, *c* = 7.6926 Å, *α* = 59.5583°, *β* = 86.0485°, and *γ* = 76.9951° (Table S2, Supporting Information). As illustrated in Figure 2g and Figure S8 (Supporting Information), after deprotonation, four imino groups in TABQ were *bi*‐chelated with square‐planar Cu ions to form 1D chains, exhibiting a slipped *π*‐stacking model (Figure 2g). The ligands in adjacent chains contacted with each other by intermolecular NH···O hydrogen bonds. A slight distortion was observed in M‐(NH)_4_ square planar structure due to the 3d^9^ orbital configuration of Cu^2+^ ions.^[^ ^22^ ^]^ The co‐existence of *π*–d conjugation, *π*–*π* stacking and hydrogen bonds may be the reason that short quasi‐1D morphologies (Figure S1, Supporting Information) rather than 1D morphologies were formed for 1D‐CuTABQ framework. While, similar to tetraoxolene ligands, such as dhbq and Cl_2_dhbq, a tris‐chelating cluster based on TABQ ligands with octahedral module can be obtained, which further be bridged by adjacent ligands into a network structure with 2D (6,3) topology.^[^ ^11^, ^17^ ^]^ As such, the lattice parameters were calculated to be *a* = *b* = 14.0976 Å and *c* = 8.970 Å with Rietveld refinement (*R*_p_ = 2.06%, *R*_wp_ = 2.66%) for 2D‐CuTABQ (Figure 2f and Table S2, Supporting Information). In 2D‐CuTABQ, each Cu ion possesses a local *D*_3_ symmetry and is surrounded by six nitrogen atoms from three chelating ligands, resulting in a trigonal cluster with octahedral geometry (Figure S9, Supporting Information). The two‐dimensional layers were stacked in an eclipsed arrangement along the *c* axis, resulting a honeycomb‐like channel (Figure 2h,i). Unlike other anionic layered structure based on Cl_2_dhbq,^[^ ^11^, ^23^ ^]^ the framework of 2D‐CuTABQ presented in an overall neutral state, in which the charge of metal ions was compensated by the in situ oxidation of the ligands. Such 2D framework resulted in the 2D morphologies (Figure 2c). High‐resolution transmission electron microscopy (HRTEM) images were carried out. HRTEM image of 1D‐CuTABQ showed a *d*‐spacing of about 4.75 Å, which agreed well with the diffraction peaks at 18.8° (Figure 2b,e). While the obvious lattice fringe (0.32 nm) in the HRTEM images of 2D‐CuTABQ was in accordance with the highest diffraction peaks at 28.2° (Figure 2d,f). These results verified the proposed structures of 1D‐CuTABQ and 2D‐CuTABQ frameworks.

Diffuse reflectance UV–vis spectroscopy was used to further probe the degree of electron delocalization and metal–ligand conjugation in the two frameworks. As shown in Figure S10 (Supporting Information), 1D‐CuTABQ exhibited a strong absorptions around 418 nm, which could be assigned to ligand‐centered (LC) *π*–π* transition. However, in comparison with free ligand, the maximum absorption was red‐shifted, indicating the electron delocalization between Cu^2+^ and TABQ in square planar structure.^[^ ^15b^ ^]^ Notably, 1D‐CuTABQ displayed a broad absorption starting from 860 nm and extended to the near‐infrared region, which could be attributed to the metal–ligand d–*π* conjugation.^[^ ^12^ ^]^ In contrast, 2D‐CuTABQ showed a relatively weak and broad absorption, which was somewhat similar with the free ligand, indicating the less electron delocalization in non‐planer octahedral unit. The broad absorbance observed in the range of 220–1000 nm could likely be assigned to the metal‐to‐ligand charge transfer (MLCT), which was also observed in other dhbq and Cl_2_dhbq based CPs.^[^ ^18b^ ^]^ In addition, the optical band gap of 1D‐CuTABQ and 2D‐CuTABQ can be estimated to be 0.80 and 1.30 eV, respectively, further confirming the different charge delocalization due to the different frameworks. Furthermore, the bulk electron conductivity of 1D‐CuTABQ and 2D‐CuTABQ were 9.7 × 10^−3^ S m^−1^ and 8.1 × 10^−5^ S m^−1^ (Figures S11 and S12, Supporting Information), respectively, higher than those of conventional insulated MOFs,^[^ ^1^, ^24^ ^]^ which is essential for batteries with high rate performance. The higher conductivity of 1D‐CuTABQ than that of 2D‐CuTABQ also indicated that the more planar structure and better conjugation of 1D‐CuTABQ than those of 2D‐CuTABQ.^[^ ^12^ ^]^ All these results suggested that the two materials consisted of Cu ions and TABQ ligands but had different coordination configurations and geometries.

### 2.2 Electrochemical Performance

**Figure 4:**
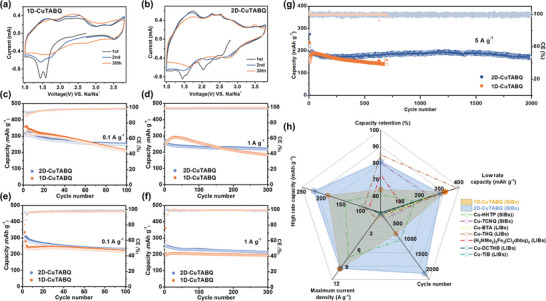
Electrochemical performance of 1D‐CuTABQ and 2D‐CuTABQ as cathodes in SIBs. a,b) The CV curves at a scan rate of 1 mV s^−1^. Cycling stability at a current density of 0.1 and 1 A g^−1^ in the electrochemical window of c,d) 1.0–3.8 V and e,f) 1.0–3.6 V, respectively. g) Cycling performance at 5 A g^−1^ in the electrochemical window of 1.0–3.8 V. h) Comparison of electrochemical performance of representative CCPs cathodes for batteries.

In order to investigate the effect of the coordination configuration and framework dimension on the electrochemical performance, cyclic voltammetry curves of two materials were first conducted in the potential range of 1.0–3.8 V (*vs* Na^+^/Na) at a current density of 1 mV s^−1^ (**Figure** **4**a,b). In the first cathodic scan, the reduction peak should be related to the reduction of both ligands (probably from the C = O groups to C–O groups) and the present Cu^2+^ (to Cu^+^), accompanying with the storage of Na ions, which will be further discussed later. It should be noted that more Cu^2+^ were present in the pristine 1D‐CuTABQ than 2D‐CuTABQ; however, only a few of the Cu^2+^ ions were reduced to Cu^+^ in 1D‐CuTABQ. The 2D structure of 2D‐CuTABQ probably facilitated the reduction of the material and the storage of Na^+^ ions. On the other hand, the following anodic scan exhibited an additional oxidation peak at about 3.5 V, which probably could be ascribed to the oxidation of pristine Cu^+^ in the materials, accompanying with the storage of anions (PF_6_^−^).^[^ ^19^, ^20^ ^]^ The more Cu^+^ in the pristine 2D‐CuTABQ resulted in more obvious oxidation peak at 3.5 V and more storage of anions. Nevertheless, 2D‐CuTABQ still displayed higher stability than that of 1D‐CuTABQ: the CV curves of 2D‐CuTABQ in subsequent cycles overlapped well with that of the 2nd cycle, indicating the high reversibility of storage of ions. Another point should be highlighted is that the redox peak at about 3.5 V was hardly observed in the electrochemical window of 1.0–3.6 V (1 m NaPF_6_ in DME, Figure S13, Supporting Information) and in order to achieve the storage of anions, a higher concentrated electrolyte (4 m NaPF_6_ in DME) was adopted here to widen the electrochemical window (for 1.0–3.8 V, similarly hereinafter, Figure S14 and S15, Supporting Information).^[^ ^25^ ^]^

The cycling performance in a voltage range of 1.0–3.8 V was further investigated at a current density of 100 mA g^−1^ by using galvanostatic scan. Among them, 1D‐CuTABQ delivered high initial discharge/charge capacities of 347.8/358.0 mAh g^−1^ (Figure 4c and Figure S16, Supporting Information), confirming three electron redox process for every coordination unit (theoretical capacity of 354 mAh g^−1^), which should be referred to two electron transfer for ligand (two carbonyl groups in every TABQ) and one electron transfer for Cu ion. However, relatively poor cycling stability was observed with a capacity of 210 mAh g^−1^ after 100 cycles and a capacity retentionof only ≈60%. When the electrochemical window was set in the range of 1.0–3.6 V, lower capacity was obtained (Figure S17, Supporting Information), though kind of oxidation of the Cu^+^ ions still existed. On the other hand, although 2D‐CuTABQ delivered relatively lower initial discharge/charge capacities of 273.9/319.6 mAh g^−1^ (Figure 4c and Figure S18, Supporting Information), the charge storage mechanism was similarly, i.e., it involved two‐electron transfer of every TABQ (two C = O groups) and one‐electron transfer reaction of Cu^2+^. Noted that the molar ratio of TABQ and Cu ions in 2D‐CuTABQ was 1.5:1 and half of the pristine Cu ions was Cu^2+^, hence the initial discharge capacity is quite close to the theoretical capacity (301 mAh g^−1^, corresponding to 3.5 electrons for Cu(TABQ)_1.5_, 3 electrons for the 1.5 TABQ and 0.5 electrons for the pristine Cu^2+^), accompanying the intercalation/extraction of sodium ions. The capacity contribution of Cu and TABQ in the first cycle was also coincident well with the reduction peaks in the CV curves. The reduction peak at about 2.5 V could be accribed to the transformation of Cu^2+^ to Cu^+^. On the other hand, the peaks at about 2.0 and 1.5 V should be all attributed to the redox reactions of C=O groups. The electron transfer number of Cu:TABQ (1:6) agreed well with the ratio of the integral areas for redox peaks of Cu and C=O groups (Figure S19, Supporting Information). On the other hand, the recharge capacity is higher than the initial discharge capacity, but slightly lower than the theoretical capacity (344 mAh g^−1^, corresponding to 4 electrons for Cu(TABQ)_1.5_, 3 electrons for the 1.5 TABQ and 1 electron for all the Cu^2+^/Cu^+^), indicating that the reduced Cu^+^ were re‐oxidized to Cu^2+^ and the pristine Cu^+^ were also oxidized to Cu^2+^. Interestingly, excellent cycling stability was observed with a capacity retention of ≈81% (247.0 mAh g^−1^) after 100 cycles and ≈74% (225.0 mAh g^−1^) after 200 cycles, which probably should be ascribed to the porosity structure of 2D‐CuTABQ that facilitated the diffusion of ions and enabled the well accommodation of Na^+^ and large sized anions. After 300 cycles at 1.0 A g^−1^, about 80% of the capacity was still maintained, which was much better than that of 1D‐CuTABQ (≈63% capacity retention) (Figure 4d). Moreover, a remarkably durable cycling performance with capacity of 170 mAh g^−1^ after 2000 cycles was obtained at 5 A g^−1^ for 2D‐CuTABQ (Figure 4g), which referred to an average capacity decay of ≈0.01% per cycle and was superior to not only 1D‐CuTABQ but also almost all of the reported MOFs as cathodes for batteries (Figure 4h and Table S3, Supporting Information).^[^ ^26^ ^]^ Of particular note is that the 1D‐CuTABQ showed an activation process at higher rate (both 1 and 5 A g^−1^), suggesting the non‐porous structure of 1D‐CuTABQ that confined the ionic diffusion and storage and at high current densities the time for mass transfer was insufficient. Furthermore, the capacity of 1D‐CuTABQ decreased rapidly afterward compared with those of 2D‐CuTABQ. The rate capability of the 2D‐CuTABQ cathode was further measured at 0.1–10 A g^−1^ (Figure S20, Supporting Information). Even at a current density as high as 10 A g^−1^, the 2D‐CuTABQ electrodes can still deliver a reversible capacity of 144 mAh g^−1^, demonstrating an excellent rate capability. Moreover, the specific capacity can be recovered to 274 mAh g^−1^, when the current density was reduced back to 0.1 A g^−1^, confirming the good reversibility of 2D‐CuTABQ cathode. As control experiment, the capacity retentions of 1D‐CuTABQ at high rates were much lower than those of 2D‐CuTABQ (Figure S21, Supporting Information). All of these results indicated that the porosity structure of 2D‐CuTABQ facilitated the diffusion and storage of ions.

Another interesting point is that the capacity, stability and rate performance of 1D‐CuTABQ in the working potential range of 1.0–3.6 V (vs Na/Na^+^) were similar to those of 2D‐CuTABQ (Figure 4e,f and Figure S22, Supporting Information). These results indicated that in the potential range of 1.0–3.6 V, storage of anions rarely occurred for both materials and hence the much better performance of 2D‐CuTABQ in the working potential range of 1.0–3.8 V suggested that the porosity structure of 2D‐CuTABQ particularly facilitated the diffusion and storage of large sized anions.

### 2.3 Charge‐Storage Mechanism

**Figure 5:**
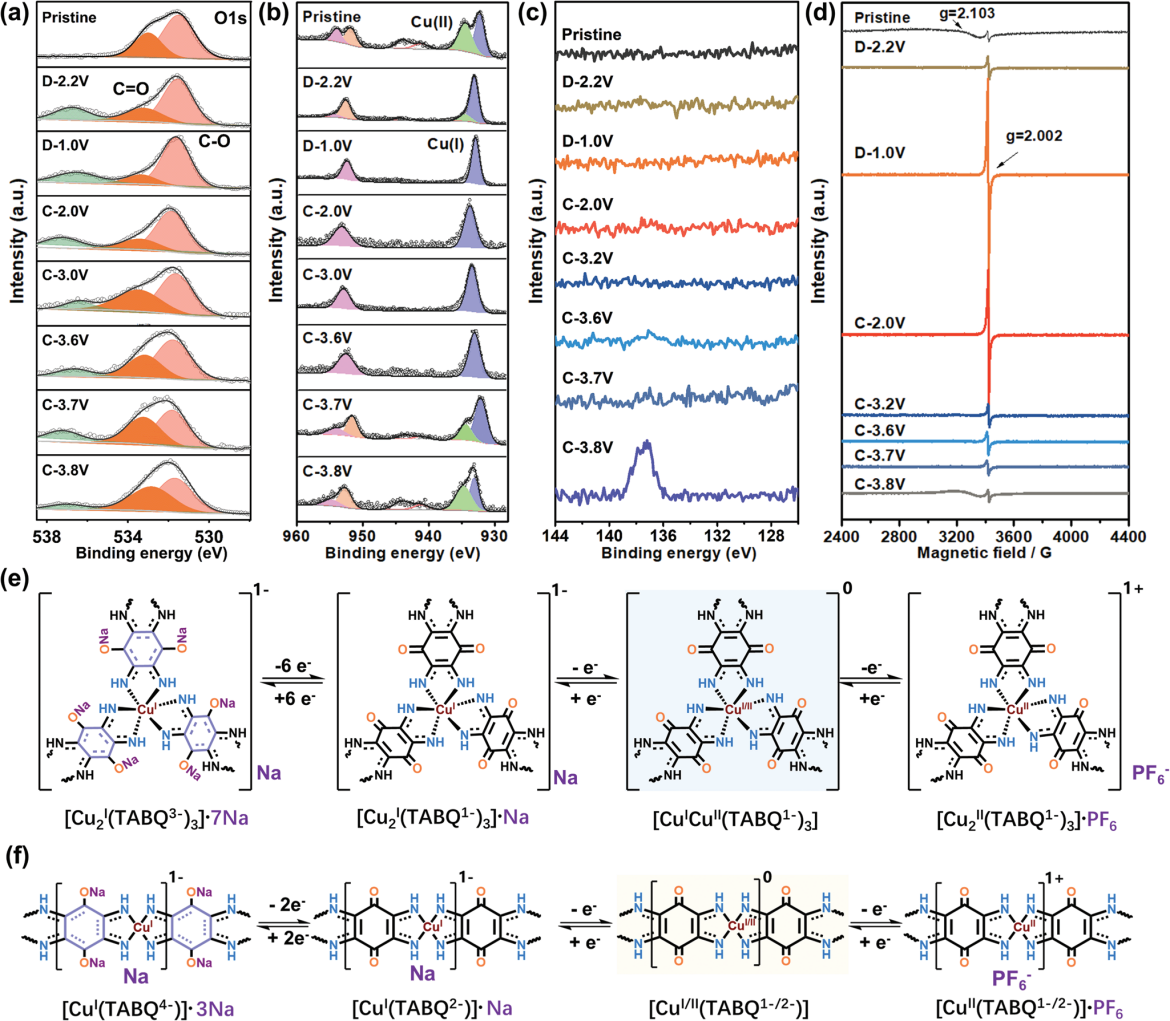
Ex situ a) O 1s, b) Cu 2p, and c) P 2p XPS spectra of the 2D‐CuTABQ electrodes were recorded at different potentials during the first cycle. d) Ex situ EPR spectra of the 2D‐CuTABQ electrodes were recorded at different potentials. e,f) Proposed reaction mechanism of 2D‐CuTABQ and 1D‐CuTABQ. The structures with colored background are the pristine states of materials. Noted that the reduction of the ligands probably occur on the carbonyl groups and hence the structures of TABQ^3−^ and TABQ^4−^ were different from the illustrated states in Figure 1.

The charge‐storage mechanism of the two materials was further conducted to better understand the difference between the two materials. From the ex situ FT‐IR spectra of 2D‐CuTABQ, the peaks at 1690 cm^−1^ that could be assigned to C=O groups vanished after reduction, alternatively with the appearance of a new absorption of C—O bond stretching at 1236 cm^−1^. Upon re‐oxidation, a reverse transformation from C–O to C=O groups could be observed, which agreed well with the ligand‐centered two‐electron reaction mechanism for quinone‐based electrodes (Figures S23 and S24, Supporting Information).^[^ ^27^ ^]^ The ex situ XPS analysis showed a similar phenomenon. As depicted in O 1s spectra, the peak intensity of C=O at ≈533.0 eV and C–O at ≈531.6 eV changed cyclically during the electrochemical reaction, which confirmed that C=O groups were involved in the redox process (**Figure** **5**a). The characteristics of benzene ring breathing bands at 1590 cm^−1^ appeared after being fully discharged to 1.0 V and almost disappeared when charged to 3.2 V, indicating the appearance of resonance structures after electron redistribution (Figure S23, Supporting Information).^[^ ^28^ ^]^ Besides, the vanished peak at 1485 cm^−1^ and blue shift C–N stretching band after discharging were observed in FT‐IR spectra, which confirmed the electron redistribution of C—N/C=N bond after being discharged. It should be noticed that the redox potential of C=N/C—N bonds in similar CCPs was at about 1.2 V or even lower than 1 V in SIBs, typically lower than the redox potential of C=O groups.^[^ ^6^, ^16^, ^29^ ^]^ In this case, after the reduction of C=O bonds in TABQ, the acceptance of electrons at C=O bonds will lower the redox potential of C=N/C—N bonds and hence the variation of C=N/C—N bonds in N 1s XPS spectra during discharge/charge process probably should be contributed to the electron redistribution rather than the direct redox reaction of C=N/C—N bonds (Figure S25, Supporting Information).^[^ ^16^, ^28^ ^]^ The FT‐IR and O 1s XPS spectra of 1D‐CuTABQ electrodes showed similar phenomena to those of 2D‐CuTABQ electrodes (Figures S26–S28, Supporting Information), revealing the similar redox activity of organic ligands with the intercalation/extraction of Na^+^. Moreover, the ex situ XPS spectra of Cu 2p in 2D‐CuTABQ showed that the signal of Cu^2+^ disappeared after discharged to 1.0 V, leading to dominated Cu^+^, and then kept as Cu^+^ until recharged to 3.6 V (Figure 5b). When it was further charged to 3.8 V, the signal of Cu^2+^ reappeared. From the integral areas of the two copper ions in XPS spectra, more than ≈70% of Cu ions were oxidized to Cu^2+^ at a cut‐off voltage of 3.8 V at first cycle, proving the recovery of pristine Cu^2+^ (50%, corresponding to the storage of Na^+^) and oxidation of partial Cu^+^ (20%, corresponding to the storage of PF_6_^−^ anions). The storage of PF_6_^−^ anions could be confirmed by the additional peak in P 2p spectra at the fully charged state of 2D‐CuTABQ electrodes (Figure 5c). The percentage of Cu^2+^ and the storage of PF_6_^−^ anions increased after more cycles (Figure S29, Supporting Information). Although the variation of Cu ions in 1D‐CuTABQ was also similarly, the transformation from Cu^2+^ to Cu^+^ after discharge and the storage of anions were relatively less and uncompleted (Figure S30, Supporting Information). These results proved the proposed mechanism and good dual‐ion storage in 2D frameworks.

Considering both the ligands and metal ions based redox reactions involved the evolution of single electrons, ex situ electron paramagnetic resonance (EPR) measurement was recorded at different charge/discharge states to further verify the charge storage mechanism. As described in Figure 5d, when being discharged to 1.0 V, the broad EPR signal of Cu^2+^ in 2D‐CuTABQ disappeared, indicating the complete reduction of metal ions, which led to the transformation of electronic configuration from *d* ^9^ (Cu^2+^) to *d* ^10^ (Cu^+^). A sharp EPR signal centered at *g* = 2.002 appeared and then significantly increased during discharge, which could be ascribed to the presence of unpaired electrons on the ligands after reduction of C=O groups to C–O^**·‐**^ radical (Figure 5d). Moreover, the recharged electrodes materials showed a decreased intensity of ligand based EPR signal at voltage below 3.2 V, and remained unchanged even when charged to 3.8 V, indicating that the redox reaction of ligands occurred at 1.0–3.2 V.^[^ ^20^ ^]^ Moreover, a broad EPR signal at *g* = 2.100 appeared again after recharge, suggesting that the redox peak above 3.2 V should be attributed to the redox reaction of Cu^2+^/Cu^+^. The EPR signal of 1D‐CuTABQ is quite similarly (Figure S31, Supporting Information). These results reconfirmed the proposed mechanism of dual ion storage by virtue of the redox of both ligands and Cu ions (Figure 5e,f).

The ex situ PXRD were recorded at different potentials to study the structure stability during cycling. As shown in Figure S32 (Supporting Information), the peaks at 7.20° (010) and 10.08° (001) have a slight left‐side shift when discharged to 1.0 V, indicating that the Na^+^ ions inserted into the space of 2D‐CuTABQ and increased the lattice constant. When it was recharged to 3.6 V, the lattice parameters returned to the initial states, indicating the reversibility of the materials. When being charged to 3.8 V, although the intercalation of large sized PF_6_^−^ anions will cause the change of the lattice parameters of the unit cell, the peaks at 23.0° and 28.3° still maintained, indicating the stability of the frameworks.^[^ ^14c^ ^]^ As such, the high electrochemical performance could be attributed to the porosity and robust structure of 2D‐CuTABQ.

### 2.4 Reaction Kinetics

The reaction kinetics were further studied to reveal the reason of good dual‐ion storage of 2D‐CuTABQ. The CV curves at different scan rates were measured to further analyze the ionic diffusion behaviors by using the equation *i* = a*ν* ^b^, where *i* was the current and *ν* was the scan rate (Figures S33–S36, Supporting Information).^[^ ^30^ ^]^ Of note, the average *b*‐value of 2D‐CuTABQ reached 0.85 at voltage range of 1.0–3.8 V, suggesting the dominated capacitive processes (Figure S33, Supporting Information). The *b*‐values of 2D‐CuTABQ were higher than those of 1D‐CuTABQ (Figure S35, Supporting Information). Furthermore, the capacitive contributions were quantitatively evaluated. The more capacitive contributions in 2D‐CuTABQ than those in 1D‐CuTABQ were observed (Figures S34 and S36, Supporting Information), indicated the high surface area and porosity of 2D‐CuTABQ could facilitate the ionic diffusion. Additionally, galvanostatic intermittent titration (GITT) was used to further evaluate the ion diffusion kinetics during the charge/discharge process (Figures S37 and S38, Supporting Information). The calculated ion diffusion coefficient of 2D‐CuTABQ within 1.0–3.8 V was always higher than those of 1D‐CuTABQ, which further proved the 2D framework contributed to the excellent dual ion storage.^[^ ^20^, ^31^ ^]^ Furthermore, the Nyquist plots showed that the 2D‐CuTABQ electrodes exhibited smaller charge transfer resistance after cycling, although 1D‐CuTABQ had higher bulk electrical conductivity (Figure S39, Supporting Information). This phenomenon may lead to fast reaction kinetics and long‐term cycle stability, even when anions storage was involved.

## 3 Conclusion

In summary, 1D and 2D frameworks of CCPs were controllably constructed by employing TABQ as ligands and copper ions as center ions. Under air atmosphere and room temperature, dianionic TABQ^2−^ ligand and Cu^2+^ ions were formed in a square coordination geometry, leading to ideal 1D chain framework. However, in a closed system at high temperature, the TABQ ligands could be further oxidized to valence of −1 (forming TABQ^1−^), and then coordinated with copper ions (half of the Cu^2+^ ions were reduced to the valence of +1) to form an octahedral coordination geometry, resulting a 2D network. The ligand could accept two electrons with intercalation/deintercalation of Na^+^ for every TABQ, probably due to the reduction of carbonyl groups rather than the C=N groups in coordination unit. Moreover, the pristine Cu^2+^ could also be reduced to Cu^+^, accompanying with the storage of Na^+^. On the other hand, the pristine Cu^+^ ions could also lose an electron to transform into Cu^2+^, leading to the storage of PF_6_^−^ anions. As a result, 2D‐CuTABQ showed a high performance of dual‐ion storage with high reversible capacity of ≈305 mAh g^−1^, good rate capability and high capacity retention (80% capacity retention for 2000 cycles at 5 A g^−1^, an average capacity decay of 0.01% per cycle), which outperformed 1D‐CuTABQ and almost all of the previously reported MOFs as cathodes for batteries. These results highlight the delicate structural control of CCPs for high performance batteries and other various applications.

## Conflict of Interest

The authors declare no conflict of interest.

## Supporting information

Supporting Information

## Data Availability

The data that support the findings of this study are available from the corresponding author upon reasonable request.
