# Supplementary material for: Framework Dimensional Control Boosting Charge Storage in Conjugated Coordination Polymers
Source: Adv Sci (Weinh). 2022 Dec 9;10(5):2205760. doi: 10.1002/advs.202205760 (PMC9929263; doi:10.1002/advs.202205760)
Supplement: Supplementary file 1 — Supporting Information [file ADVS-10-2205760-s001.pdf]

ADVANCED SCIENCE
Open Access

Supporting Information

for *Adv. Sci.*, DOI 10.1002/advs.202205760

Framework Dimensional Control Boosting Charge Storage in Conjugated Coordination Polymers

*Kun Fan, Cheng Fu, Yuan Chen, Chenyang Zhang, Guoqun Zhang, Linnan Guan, Minglei Mao, Jing Ma, Wenping Hu and Chengliang Wang**

Supporting Information

Framework Dimensional Control Boosting Charge Storage in Conjugated Coordination Polymers

Kun Fan, Cheng Fu, Yuan Chen, Chenyang Zhang, Guoqun Zhang, Linnan Guan, Minglei Mao, Jing Ma, Wenping Hu and Chengliang Wang[*]

Dr. K. Fan, Dr. Y. Chen, C. Zhang, G. Zhang, L. Guan, Prof. Dr. M. Mao, Prof. Dr. C. Wang

School of Optical and Electronic Information, Wuhan National Laboratory for Optoelectronics (WNLO), Optics Valley Laboratory, Huazhong University of Science and Technology, Wuhan 430074, China

E-mail: clwang@hust.edu.cn

C. Fu, Prof. Dr. J. Ma

School of Chemistry and Chemical Engineering, Nanjing University, Nanjing 210093, China.

Prof. Dr. W. Hu

Tianjin Key Laboratory of Molecular Optoelectronic Sciences, Department of Chemistry, School of Sciences, Tianjin University, Tianjin 300072, China.

Dr. K. Fan, Dr. Y. Chen, Prof. Dr. C. Wang

Wenzhou Advanced Manufacturing Technology Research Institute, Huazhong University of Science and Technology, Wenzhou 325035, China

Experimental section

Synthesis of 1D-CuTABQ. In a typical synthesis, $Cu(NO_3)_2{\cdot}3H_2O$ (241.5 mg, 1 mmol) was first dissolved in 30 mL DMSO. Then subsequently, 3mL of concentrated aqueous ammonium hydroxide (~14 M) was added. The fresh 2,3,5,6-tetraaminobenzoquinone (TABQ, 1 mmol, 168 mg) was dissolved in DMSO (30 mL) and then dropped into the copper solution under stirring. The reaction was further stirred at 500 rpm for 12h at room temperature. The resulting precipitate was isolated by filtration. The powder was subsequently washed with DMSO (50 mL × 2), acetone (25 mL × 2) and dried in 80 °C oven for further characterization. Yield: 244 mg. Elemental analysis calculated for **1D-CuTABQ**, Anal. Calcd for $Cu(C_6H_4N_4O_2)(C_2H_6OS)_{0.025}(H_2O)$: C, 29.34%; H, 2.50%; N, 22.62%, S, 0.32%, O, 19.54%, Cu, 25.66%. Found: C, 29.73%; H, 2.44%; N, 21.92%, S, 0.37%, O, 20.42%, Cu, 25.43%.

Synthesis of 2D-CuTABQ. In a typical synthesis, the fresh 2,3,5,6-tetraaminobenzoquinone (TABQ, 0.3 mmol, 50.4 mg) was dissolved in DMSO (12 mL) in a 38 ml vial. Then $Cu(NO_3)_2{\cdot}3H_2O$ (108.7 mg, 0.45 mmol) in 6 mL DMSO was added slowly. The vial was tightly sealed and then transferred into a 120 ^{o}C oven for 72 h. A black precipitate was obtained by vacuum filtration and washed with water. Yield: 72 mg. Anal. Calcd for $Cu(C_6H_4N_4O_2)_{1.5}(C_2H_6OS)_{0.5}(H_2O)$: C, 32.74%; H, 3.02%; N, 22.91%, S, 4.37%, O, 19.63%, Cu, 17.32%. Found: C, 34.28%; H, 2.55%; N, 20.25%, S, 3.50%, O, 20.93%, Cu, 17.58%.

Materials and physical measurements. All starting materials were obtained from commercial sources without further purification. Scanning electron microscopy (SEM) and transmission electron microscopy (TEM) images were recorded by a Gemini 300 (Zeiss) electron microscopy and JEOL 2100 instrument, respectively. Powder X-ray diffraction (PXRD) data were collected by X' Pert3 Powder instrument with a Cu Kα X-ray radiation (λ = 1.5406 Å) at room temperature. The Fourier transformed infrared spectroscopy (FTIR) were measured on a Bruker ALPHA spectrometer in the range of 400-4000 cm^{-1}. N_2 adsorption/desorption isotherms were measured using a

BELSORP-max instrument (BEL Japan, Inc.). Thermogravimetric analyses (TGA) were performed under a flow of nitrogen in the temperature range 35 - 800 °C at a heating rate of 10 °C / min using a Pyris1 thermogravimetric analyzer. XPS spectrum was collected on a Thermo Fisher Esca Lab 250Xi using a monochromic Al X-ray source ($h\nu$ = 1486.6 eV). Elemental analyses for C, H, N, O and S were obtained with a Vario Micro Cube Elemental Analyzer. The content of metal ions was determined by the ICP-OES (Agilent 730). X-band EPR spectra were acquired on a Bruker EMS nano spectrometer at room temperature.

Computational method: The geometry optimizations and frequency calculations were performed using the B3LYP functional[1] with 6-311G(d, p) basis set[2], as implemented in Gaussian 16[3]. The electrostatic potential (ESP) surfaces were mapped with the Multiwfn 3.8 program[4] and Visual Molecular Dynamics (VMD) software[5].

The geometry optimization was carried out with the periodic boundary condition (PBC) and the projected augmented wave (PAW) formalism of density functional theory (DFT) in the Vienna Ab initio Simulation Package (VASP) code. [6] The generalized gradient approximation (GGA) within Perdew-Burke-Ernzerhof (PBE) form was employed to characterize the exchange-correlation effect.[7] Hubbard U correction in the form of GGA+U was applied to compute on Cu (U = 2.5 eV, J = 0 eV) surfaces.[8] Semiempirical DFT-D3 correction via Grimme's scheme was used to describe correctly van der Waals interactions.[9] The cutoff energy for plane-wave expansion was taken as 450 eV. The k-point sampling of Brillouin zone was performed by using a Monkhorst-Pack grid of 6 × 5 × 4 for **1D-CuTABQ** and 3 × 2 × 11 for **2D-CuTABQ**.[10] All geometries were relaxed until the Hellmann-Feynman force on each atom was less than 0.02 eV/ Å, and the convergence criteria for total energy was 10^{-5} eV.

Electrochemical Measurements. Before the electrochemical study, each sample was dried at 120 °C under vacuum for 12h. The working electrodes for sodium ions batteries were prepared by mixing the as-prepared active materials, super P carbon

black, polyvinylidene fluoride (PVDF) at a weight ratio of 6:3:1, using N-methyl-2-pyrrolidone (NMP) as a homogenizer. Then the slurry was coated onto Al foil using a doctor blade and dried 12h in vacuum at 80 °C. The coin-type cells were assembled in an Ar-filled glove box with water and oxygen levels under 1.0 ppm. The separator was Celgard polypropylene (PP) membrane and glass fiber membrane (Whatman, GF/B) with a diameter of 19.0 mm, and the counter electrode was metallic Na (diameter of 14.0 mm). The electrolyte applied in the cells was 1 M $NaPF_6$ or 4 M $NaPF_6$ dissolved in dimethyl ether (DME). The half-cells were galvanostatically cycled in different voltage ranges on the LANHE-CT2001A test system (Wuhan, China) at room temperature. Cyclic voltammetry (CV) and Electrochemical Impedance Spectroscopy (EIS) were performed by BioLogic VMP3 potentiostat. For ex-situ experiments, the half-cells were cycled to the desired state of charge at a current density of 0.1 A g^{-1} and then disassembled in an argon-filled glove box.

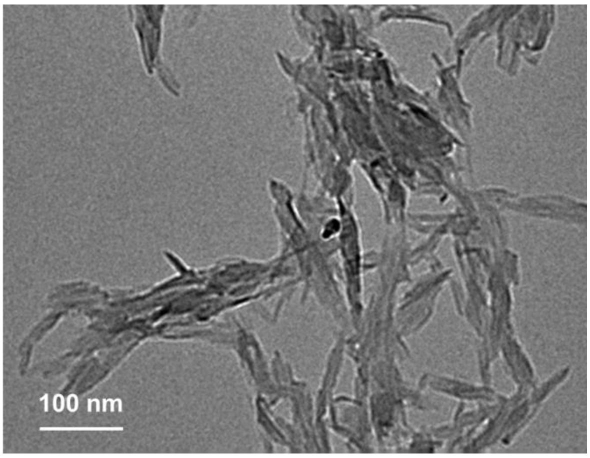

Figure S1. TEM image of bulk **1D-CuTABQ**.

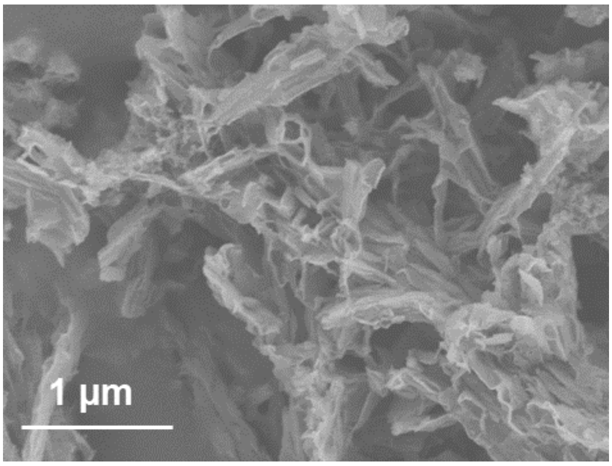

Figure S2. SEM image of bulk **2D-CuTABQ**.

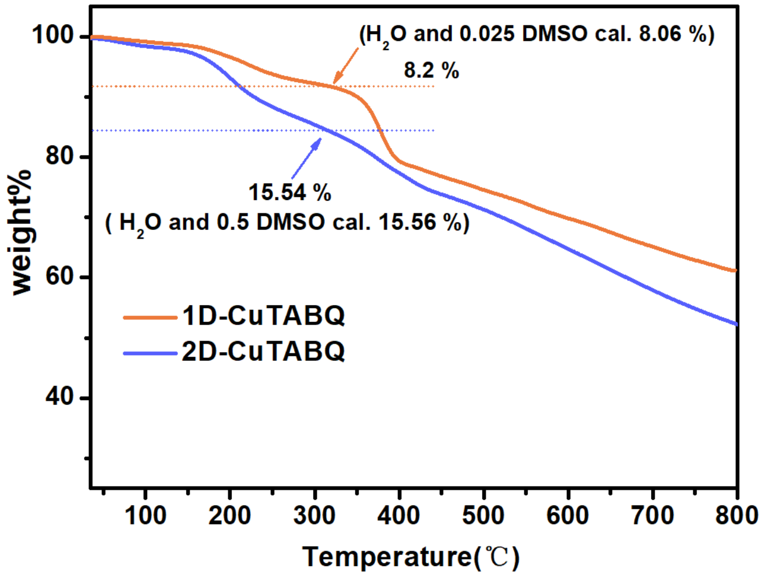

Figure S3. TGA curves of **1D-CuTABQ** and **2D-CuTABQ**.

Table S1. Experimental and theoretical elemental analysis of **1D-CuTABQ** and **2D-CuTABQ**. C, H, N, S and O content were obtained by elemental analysis and Cu content was measured by ICP-OES. The ratio of Cu^{2+} and N was approaching 1:4 in **1D-CuTABQ**. The ratio of Cu^{2+} and N was about 1:6 in **2D-CuTABQ**. The numbers of solvent molecules were inferred from the elemental analysis and confirmed by thermal analyses.

Sample	Proposed Chemical Formula	C (wt%)	H (wt%)	N (wt%)	S (wt%)	O (wt%)	Cu (wt%)
**1D-CuTABQ**	$Cu(C_6H_4N_4O_2)(DMSO)_{0.025}(H_2O)$	29.73	2.44	21.92	0.37	20.42	25.43
		Atomic Ratio					
		Found	Cu:C:H:N:O = 1:6.18:6.09:3.9:3.19				
		Theoretical	Cu:C:H:N:O = 1:6.05:6.15:4.0:3.02				
**2D-CuTABQ**	$Cu(C_6H_4N_4O_2)_{1.5}(DMSO)_{0.5}(H_2O)$	34.28	2.55	20.25	3.50	20.93	17.58
		Atomic Ratio					
		Found	Cu:C:H:N:O = 1:10.3:9.2:5.2:4.7				
		Theoretical	Cu:C:H:N:O = 1:10.0:11 :6.0 :4.5				

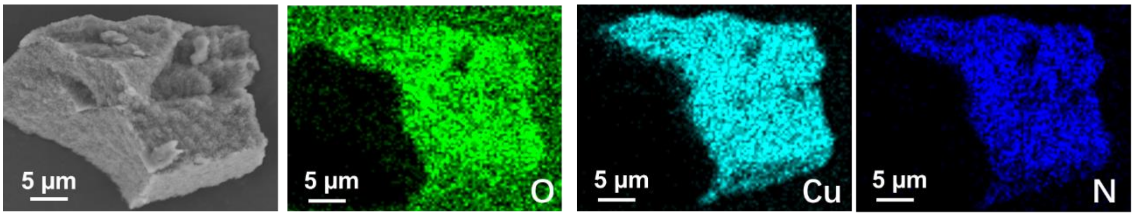

Figure S4. Energy dispersive X-ray spectrum (EDS-mapping) of **1D-CuTABQ**, which indicates that the elements are evenly distributed in the material.

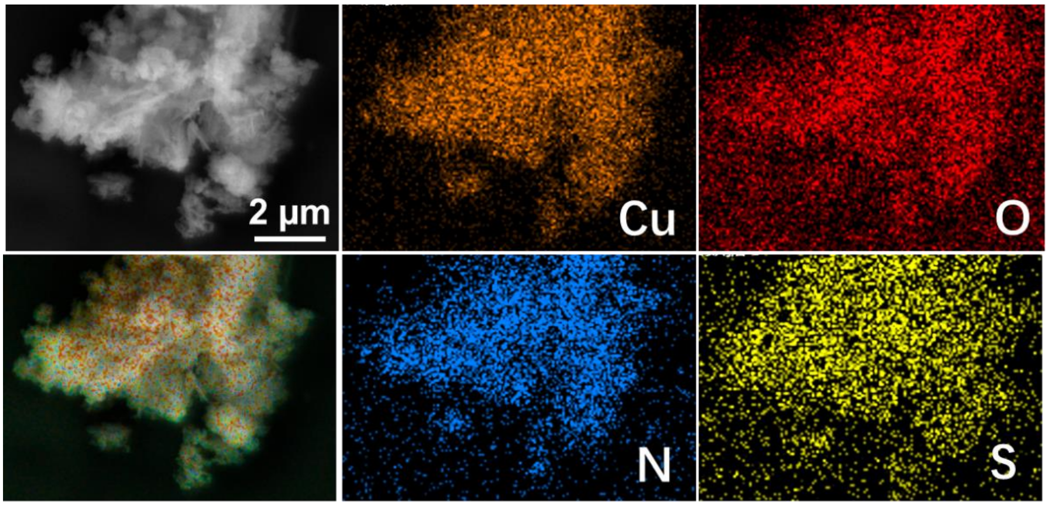

Figure S5. Energy dispersive X-ray spectrum (EDS-mapping) of **2D-CuTABQ**, which indicates that the elements are evenly distributed in the material.

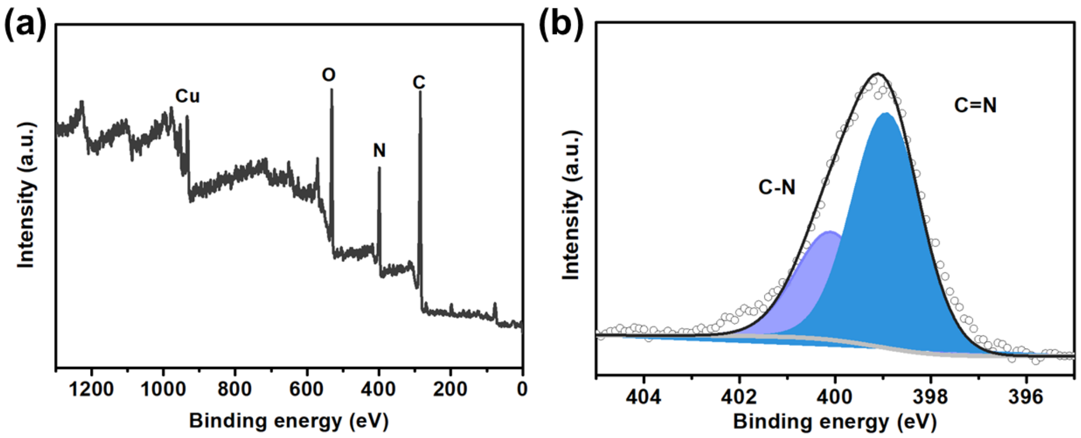

Figure S6. XPS analysis of full energy spectrum for (a) all elements and (b) N 1s spectra of **2D-CuTABQ**. Both the signal of C-N single bonds (~400.1 eV) and C=N double bonds (~398.9 eV) are coexistence in the compound.

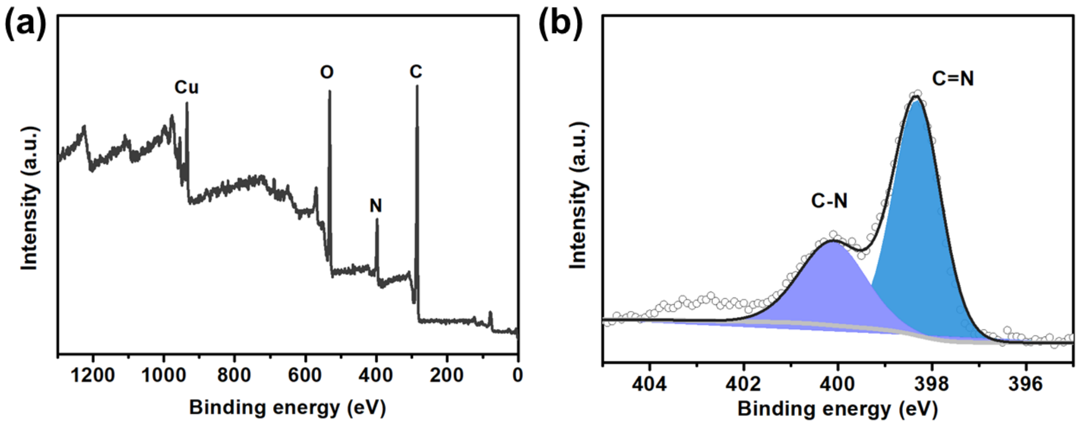

Figure S7. XPS analysis of full energy spectrum for (a) all elements and (b) N 1s spectra of **1D-CuTABQ**. Both the signal of C-N single bonds (~400.1) and C=N double bonds (~398.3 eV) are coexistence in the compound.

Table S2. Crystallographic data, powder X-ray collection conditions, and Rietveld refinement results of **1D-CuTABQ** and **2D-CuTABQ**.

Name	**1D-CuTABQ**	**2D-CuTABQ**
Chemical formula	$Cu(C_6H_4N_4O_2)$	$Cu_2(C_6H_4N_4O_2)_3$
Crystal system	Triclinic	Trigonal
Space group	*P*1	*P*-31m
a/Å	6.3792	14.0976
b/Å	7.7279	14.0976
c/Å	7.6926	8.970
α/°	59.5583	90
β/°	86.0485	90
γ/°	76.9951	120
R_p	1.77%	2.06%
R_{wp}	2.40%	2.66%

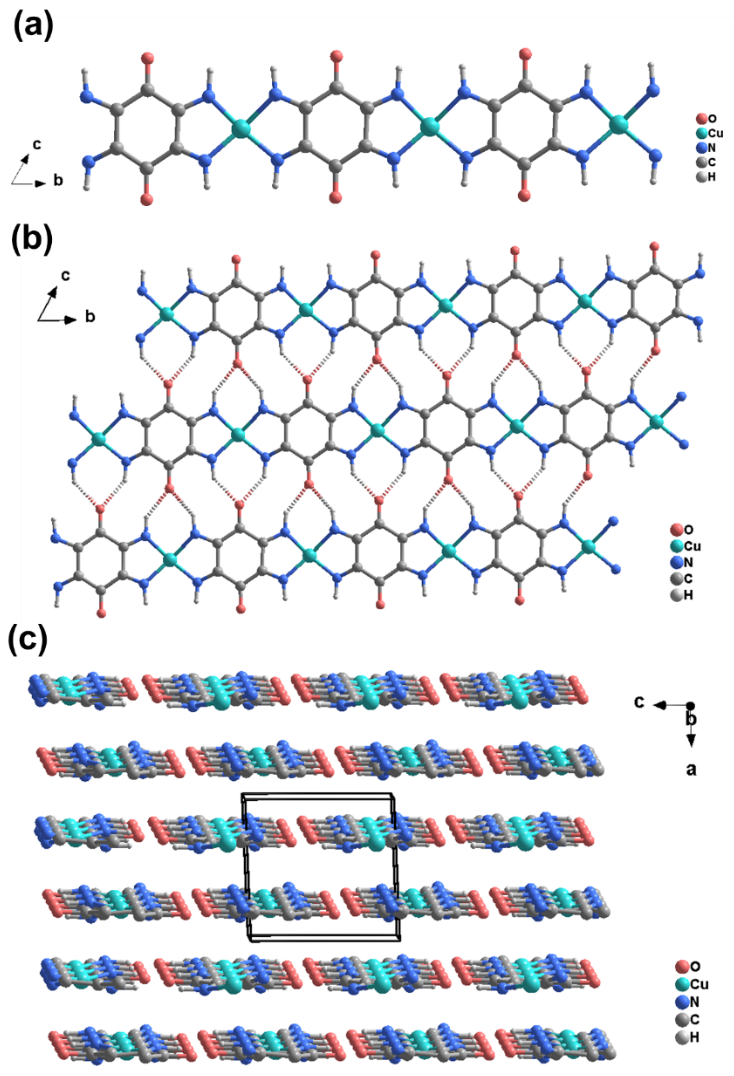

Figure S8. (a) One chain in compound **1D-CuTABQ** runs along the *b*-axis. (b) The hydrogen bonding between −NH and C=O in adjacent chains (dashed lines). (c) A slipped π-stacking model of the chains. The optimized unit cell of 1D-CuTABQ included 34 atoms, forming an eclipsed stacking model by chains extending along the b axis. There were hydrogen bonding interactions between the O atoms of carbonyl groups and H atoms of coordinated imino groups. The detailed crystallographic information was listed in the Table S2.

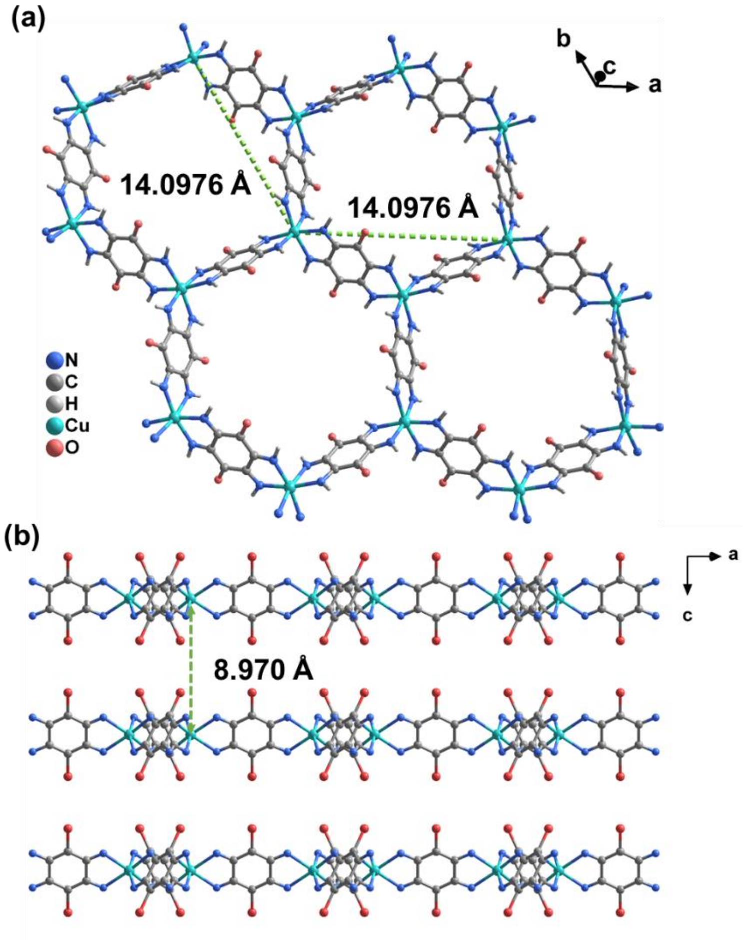

Figure S9. (a) The 2D (6,3) topology structures were simulated according to the previous reported structure and further optimized by density functional theory (DFT) calculations.[11] (b) The layers are eclipsed along the c axis with a distance of 8.970 Å.[12] The lattice solvent molecules were trapped in the space between the two layers, although it is hard to decide the exact atomic positions by Rietveld refinement. The numbers of solvent molecules were confirmed by elemental analyses and thermogravimetric analysis (TGA).

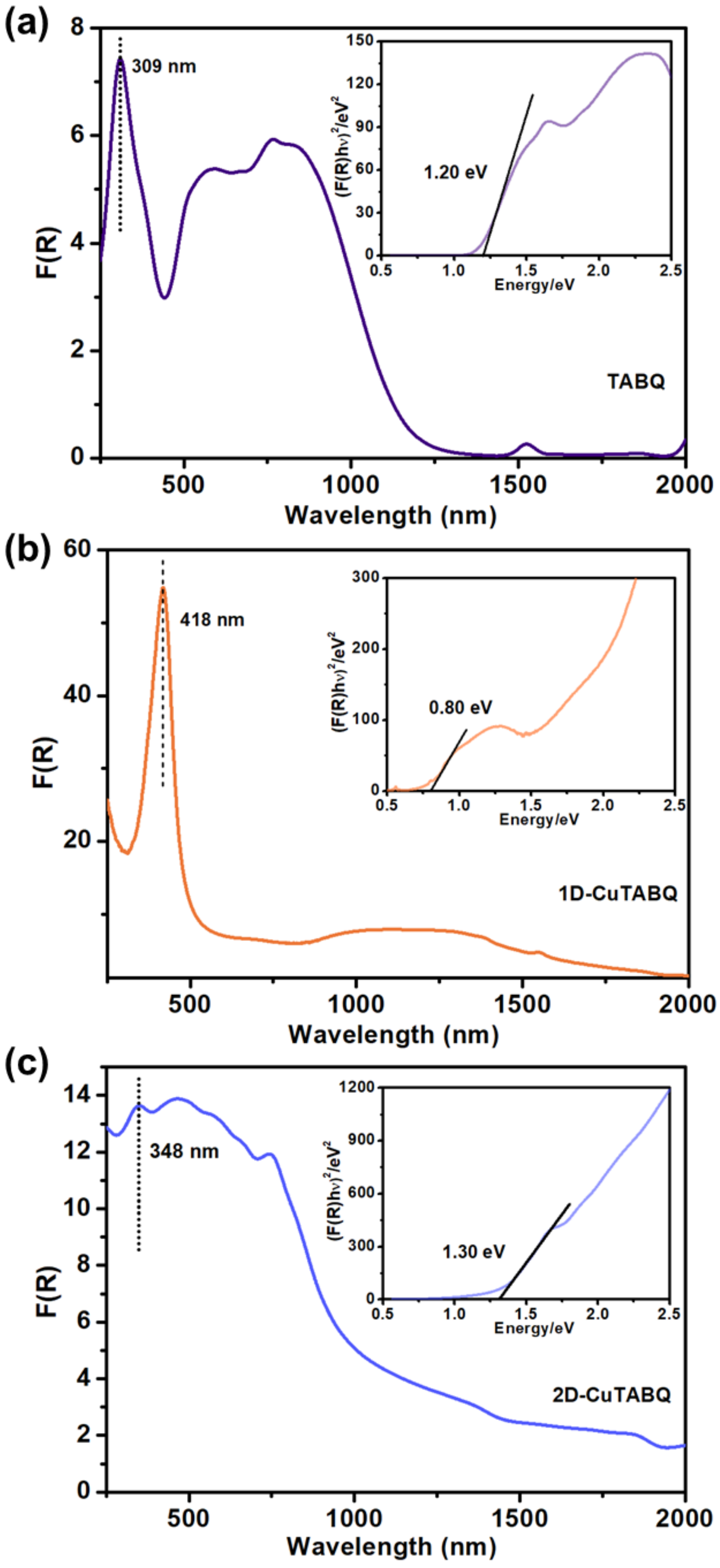

Figure S10. DRUV-vis spectra and Tauc plots of (a) TABQ, (b) 1D-CuTABQ, and (c) 2D-CuTABQ.

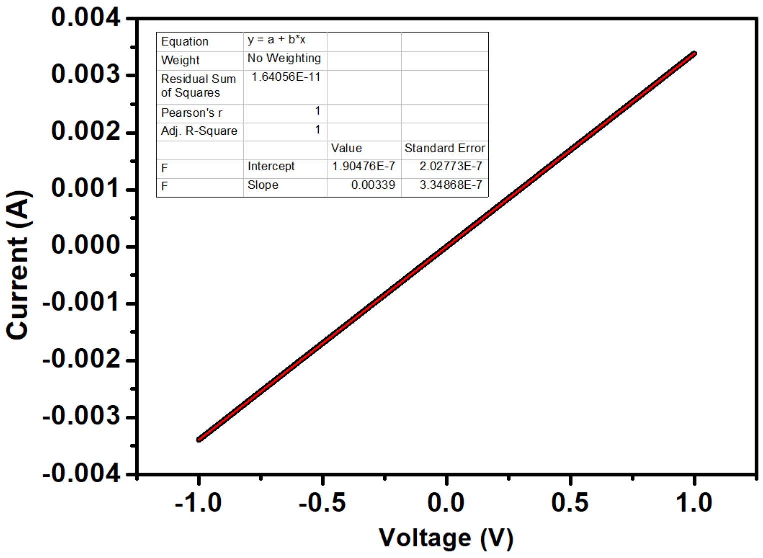

Figure S11. Linear I-V curves of **1D-CuTABQ**, giving the conductivity value of 9.3×10^{-3} S/m. The electrical conducting was calculated by σ=G *L*/*A*, where the G=0.00339 S is electric conductance, L=0.031cm is the length of the pressed pellet, A=1.13 cm^2 is cross-sectional area of the pressed pellet.

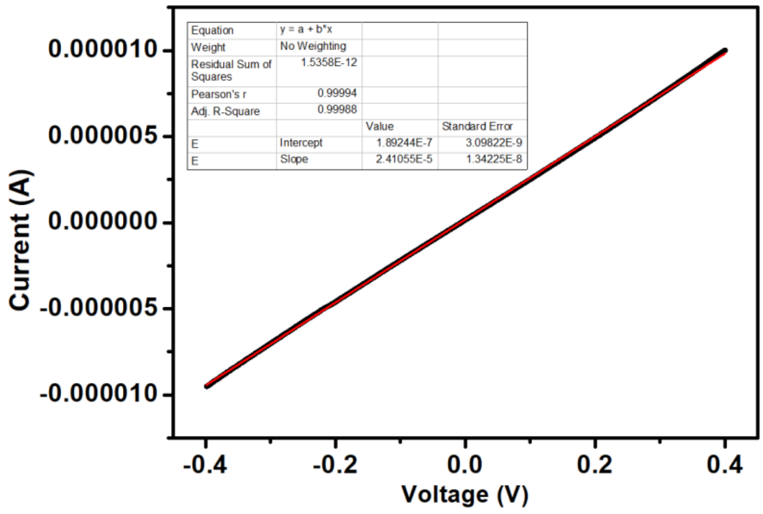

Figure S12. Linear I-V curves of **2D-CuTABQ**, giving the conductivity value of 8.1×10^{-5} S/m. The electrical conducting was calculated by σ=G *L*/*A*, where the G=2.41×10^{-5} S is electric conductance, L=0.038 cm is the length of the pressed pellet, A=1.13 cm^2 is cross-sectional area of the pressed pellet.

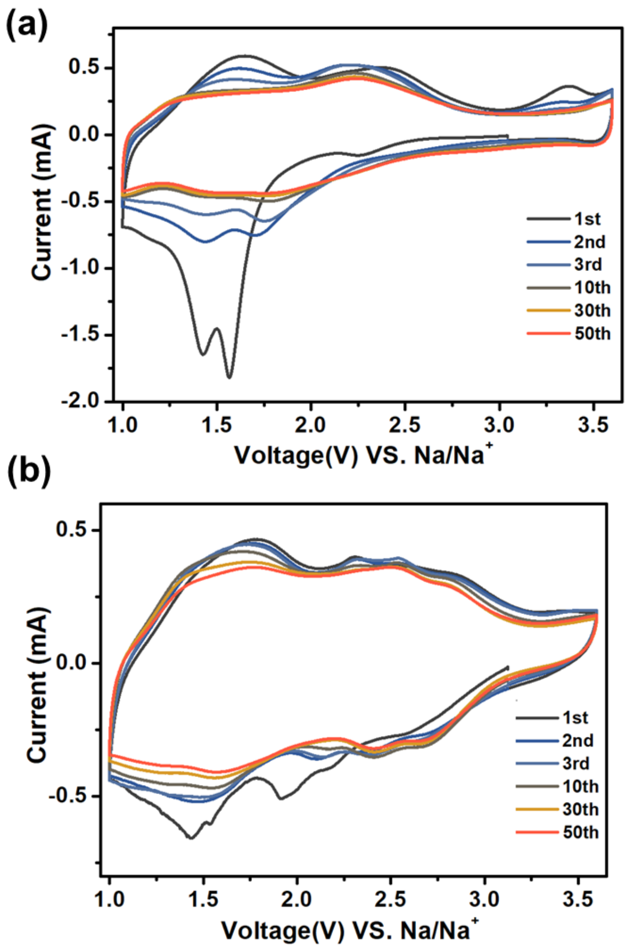

Figure S13. (a) Cyclic voltammetry curves of **1D-CuTABQ** and **2D-CuTABQ** at a scan rate of 1 mV s^{-1} within the voltage range of 1.0-3.6V.

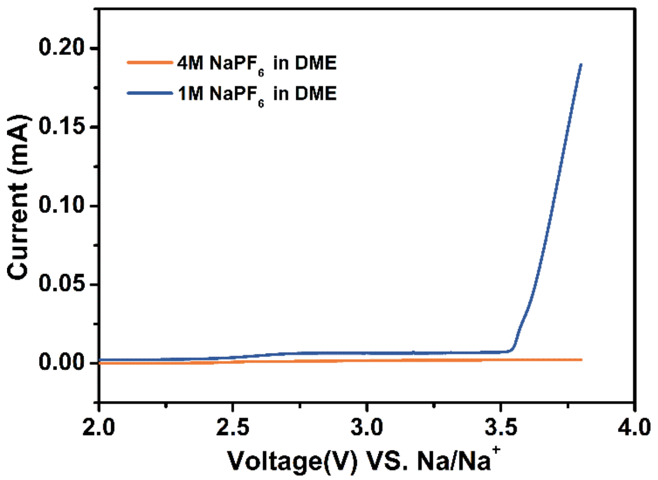

Figure S14. LSV comparison of different electrolytes. The low concentration electrolytes showed relatively poor electrochemical stability against oxidation.

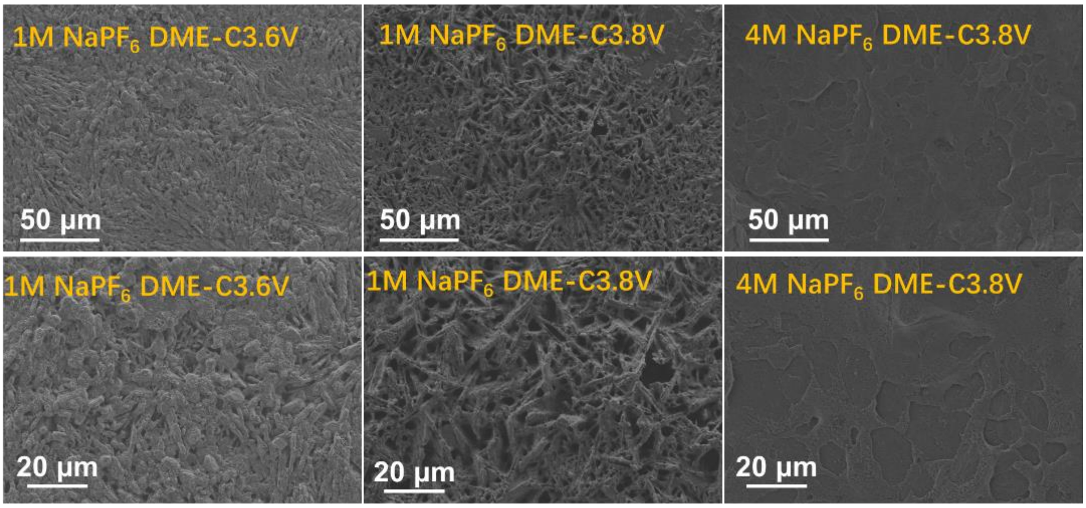

Figure S15. The SEM images of Na anodes with different potentials during cycling at a current density of 0.1 A g^{-1} when using different electrolytes. The highly concentrated electrolyte could benefit the formation of a dense and uniform SEI layer on the surface of Na metal, thereby effectively suppressing the formation of sodium dendrites and thus elevating electrochemical stability of the cells even at higher voltage.

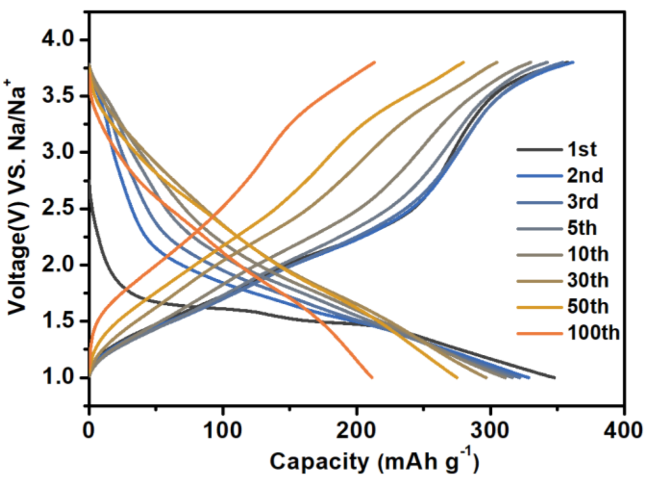

Figure S16. Discharge-charge voltage profiles of **1D-CuTABQ** at a current of 0.1 A g^{-1} within the voltage range of 1.0-3.8V.

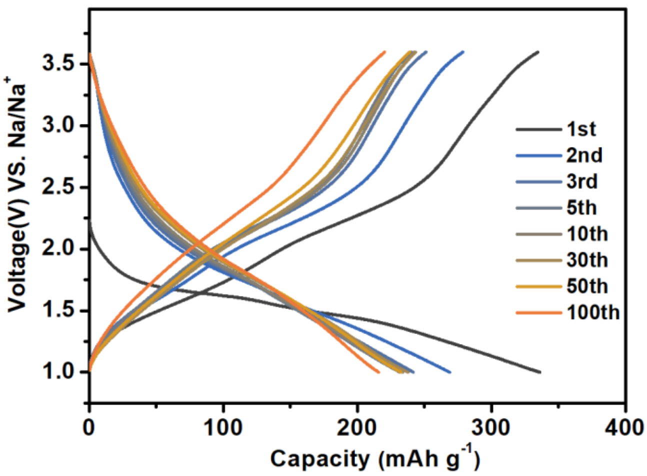

Figure S17. Discharge-charge voltage profiles of **1D-CuTABQ** at a current of 0.1 A g^{-1} within the voltage range of 1.0-3.6V.

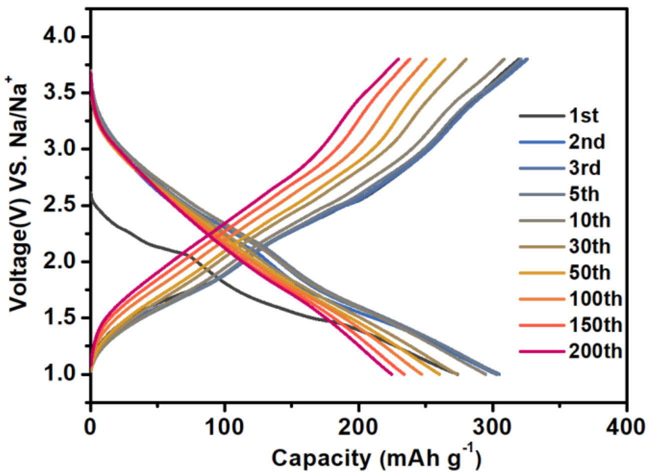

Figure S18. Discharge-charge voltage profiles of **2D-CuTABQ** at a current of 0.1 A g^{-1} within the voltage range of 1.0-3.8V.

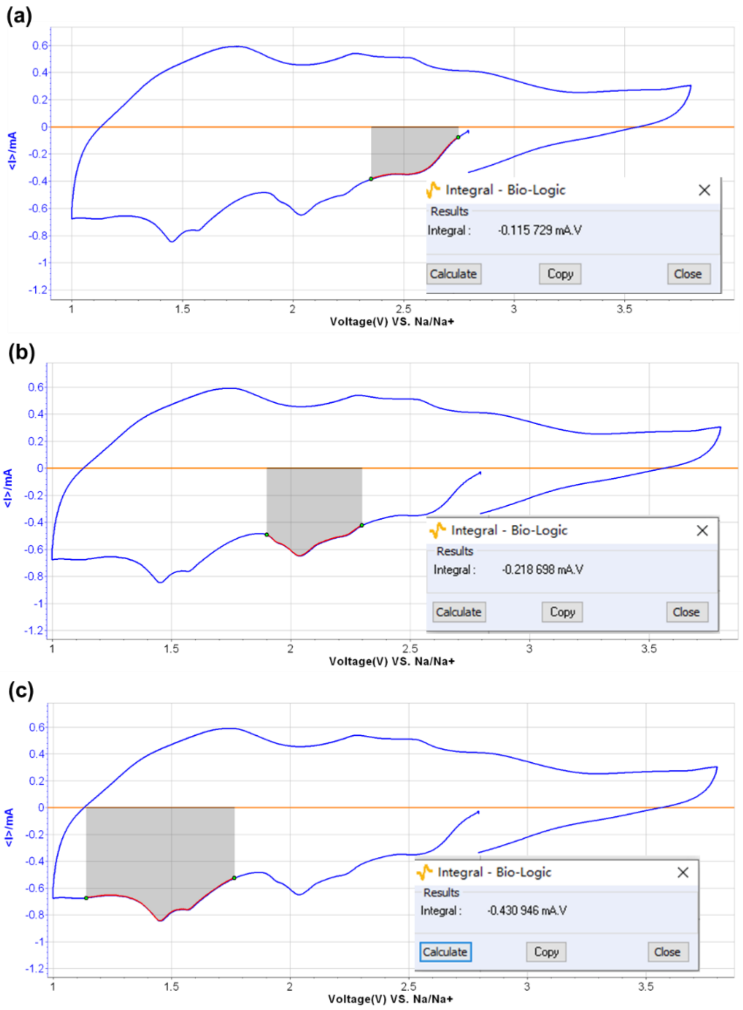

Figure S19. The area integral for three reduction peaks of 2D-CuTABQ in the first cycle.

Table S3. Electrochemical performance of representative MOFs and CCPs-based cathode materials for LIBs and SIBs.

Electrode	Battery type (Operation voltage window (V))	Active sites	Low-rate capacity (mAh g^{-1}) (Current (mA g^{-1} or C))	Reversible capacity (mAh g^{-1}) (Current (mA g^{-1} or C), cycle number)	Ref.
MIL-53	Li-ion (1.5-3.5)	Fe^{2+}/Fe^{3+}	~80 (0.1C)	~70 (0.025C, 50)	[13]
Cu(2,7-AQDC)	Li-ion (1.7-4.0)	Organic ligand Cu^{2+}/Cu^{+}	147 (/)	105 (/, 50)	[14]
MIL-47	Li-ion (1.6-3.5)	V^{3+}/V^{4+}	118 (10)	82 (10,50)	[15]
Cu-TCA	Li-ion (1.4-4.3)	Organic ligand Cu^{2+}/Cu^{+}	102 (50)	45.1 (50,200)	[16]
Mn_7(2,7-AQDC)$_6$(2,6-AQDC)(DMA)$_6$	Li-ion (1.4-4.5)	Organic ligand Mn^{2+}/Mn^{3+}	205 (/)	90 (/,50)	[17]
$(NBu_4)[Mn^{II}Cr^{III}(Cl_2An)_3]$	Li-ion (2.2-3.4)	Organic ligand Mn^{2+}/Mn^{3+}	80 (5)	62 (5,10)	[18]
$Fe_2(DFc)_3$	Li-ion (2.0-4.2)	Organic ligand Fe^{2+}/Fe^{3+}	172 (50)	96 (500, 400)<br>70 (2000, 10000)	[19]
Li_2-Mn-DOBDC	Li-ion (2.0-4.0)	Organic ligand Mn^{2+}/Mn^{3+}	70 (0.1C)	60 (2C, 100)	[20]
Ni-DI	Li-ion (2.0-4.5)	Organic ligand	155 (10)	45 (250, 300)	[21]
CuPcOH	Li-ion (2.0-4.4)	Organic ligand	128 (13)	55 (130, 200)	[22]
Cu-HHTP	Li-ion (1.7-3.5)	Cu^{2+}/Cu^{+}	~105 (~95.6)	~60 (~95.6, 100)<br>~85 (~1912, 500)	[23]
Cu-BTA	Li-ion (1.5-4.1)	Organic ligand Cu^{2+}/Cu^{+}	262 (50)	25 (50, 25)<br>50 (1000, 150)	[24]
$[CuDCTHB(DMF)_2]_n$	Li-ion (1.5-3.6)	Organic ligand Cu^{2+}/Cu^{+}	268 (30)	150 (30, 17)<br>90 (120, 45)	[25]
$Co_{0.56}Ni_{0.44}$-DI	Li-ion (1.0-3.5)	Organic ligand	248 (100)	~200 (100, 20)	[26]
Cu-THQ	Li-ion (1.2-4.0)	Organic ligand Cu^{2+}/Cu^{+}	387 (50)	~290 (50, 100)<br>~93(1000, -)	[27]
Cu-THQ	Li-ion (1.3-2.6)	Organic ligand	153 (100)	122 (100, 50)<br>~87 (1000, 1000)	[28]
$(H_2NMe_2)_2Fe_2(Cl_2dhbq)_3$	Li-ion (1.8-4.2)	Organic ligand Fe^{2+}/Fe^{3+}	198 (20)	~147 (40, 50)<br>~120 (150, 15)	[29]
Cu-BHT	Li-ion (1.5-3.0)	Organic ligand	175 (300)	~130 (300, 500)<br>~100 (1000, 500)	[30]
Fe_2(dobpdc)	Na-ion (2.0-3.65)	Organic ligand Fe^{2+}/Fe^{3+}	114 (7)	90 (140, 50)	[31]
Cu-TCNQ	Na-ion (2.0-4.1)	Organic ligand Cu^{2+}/Cu^{+}	255 (20)	~200 (50, 200)	[32]

P(THBQ-Al)	Na-ion (1.5-3.0)	Organic ligand	113 (20)	~105 (20, 100)	[33]
Ni-TTO	Na-ion (1.2-3.2)	Organic ligand	155 (100)	130 (100, 100)<br>104 (1000, 1000)	[34]
Cu-HHTP	Na-ion (1.0-3.5)	Organic ligand<br>Cu^{2+}/Cu^{+}	~200 (100)	120 (100, 250)<br>~100 (1000, 600)	[35]
Zn-HHTP	Na-ion (1.0-3.5)	Organic ligand	~150 (100)	135 (100, 1000)<br>~81 (1000, 5000)	
**1D-CuTABQ**	**Na-ion<br>(1.0-3.6)**	**Organic ligand**	**241.8 (100)**	**~193 (1000, 300)<br>~115 (5000, 1500)**	**This work**
	**Na-ion<br>(1.0-3.8)**	**Organic ligand<br>Cu^{2+}/Cu^{+}**	**328.6 (100)**	**~184 (1000, 300)<br>~140 (5000, 695)**	
**2D-CuTABQ**	**Na-ion<br>(1.0-3.6)**	**Organic ligand<br>Cu^{2+}/Cu^{+}**	**296.9 (100)**	**~212 (1000, 300)<br>~137 (5000, 1500)**	**This work**
	**Na-ion<br>(1.0-3.8)**	**Organic ligand<br>Cu^{2+}/Cu^{+}**	**305.1 (100)**	**~220 (1000, 300)<br>~170 (5000, 2000)**	

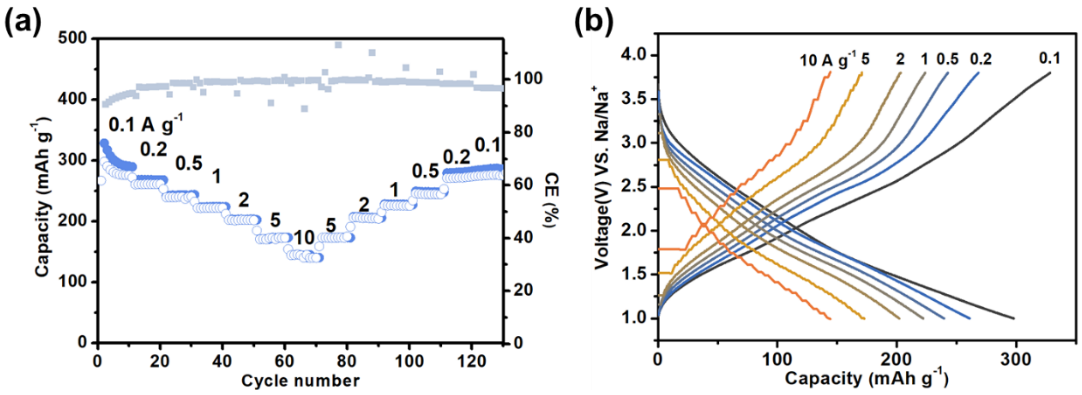

Figure S20. (a) Rate capability and (b) Typical voltage profiles of **2D-CuTABQ** electrodes for rate performance within the voltage range of 1.0-3.8V.

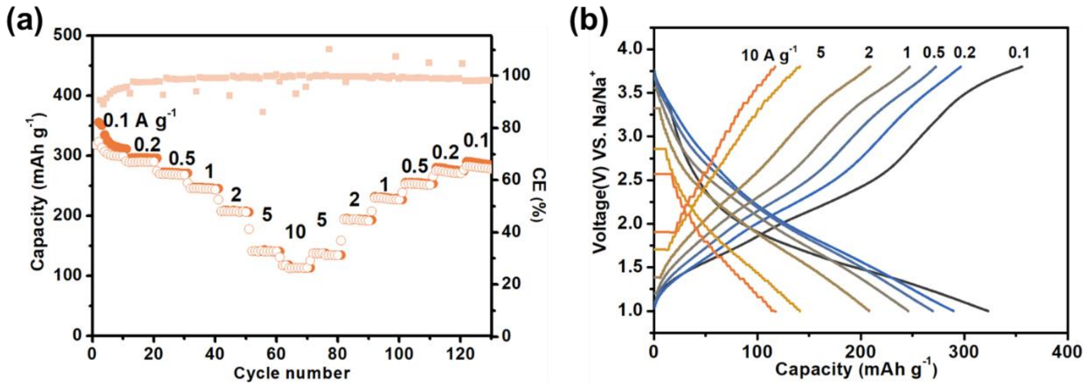

Figure S21. (a) Rate capability and (b) Typical voltage profiles of **1D-CuTABQ** electrodes for rate performance within the voltage range of 1.0-3.8V.

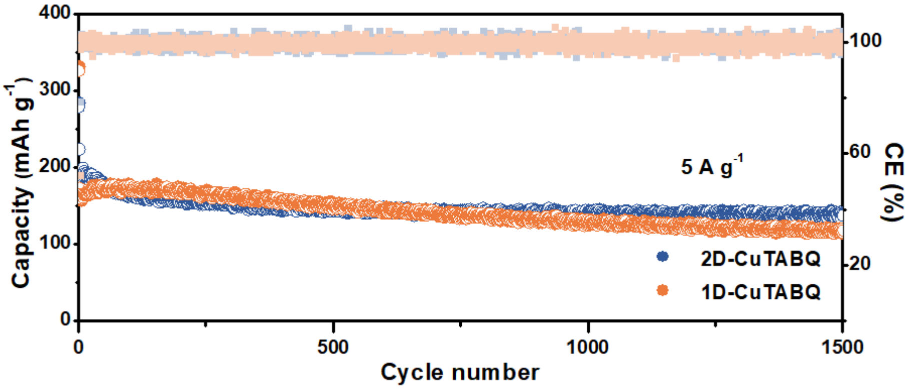

Figure S22. Cycling performance of **1D-CuTABQ** and **2D-CuTABQ** electrodes at a current density of 5 A g^{-1} within the voltage range of 1.0-3.6V.

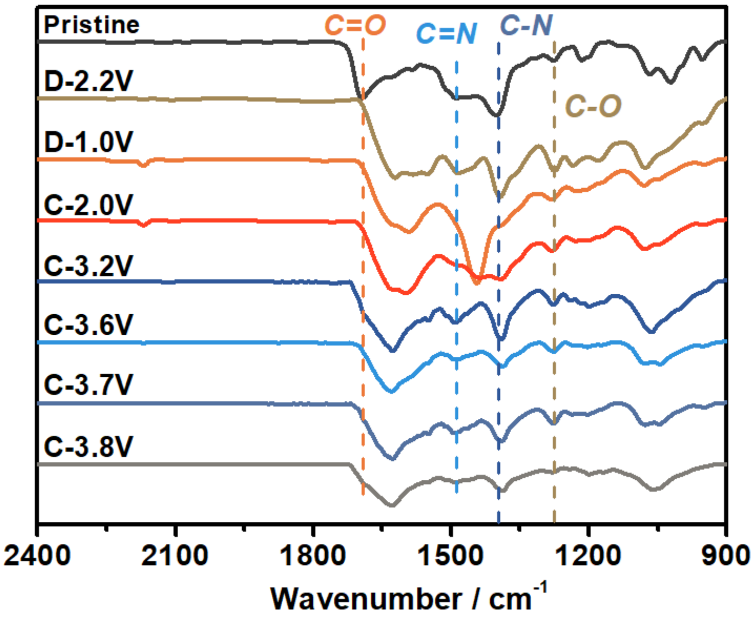

Figure S23. Ex-situ FTIR spectra of the **2D-CuTABQ** electrode were recorded at different potentials during cycling.

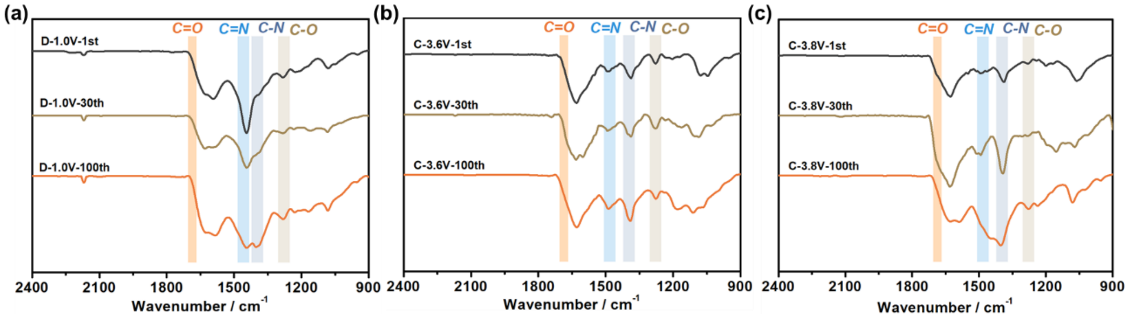

Figure S24. Ex situ FT-IR spectra of **2D-CuTABQ** electrode recorded after long-term cycling (30 cycles at 0.1 A g^{-1} and 100 cycles at 1 A g^{-1}).

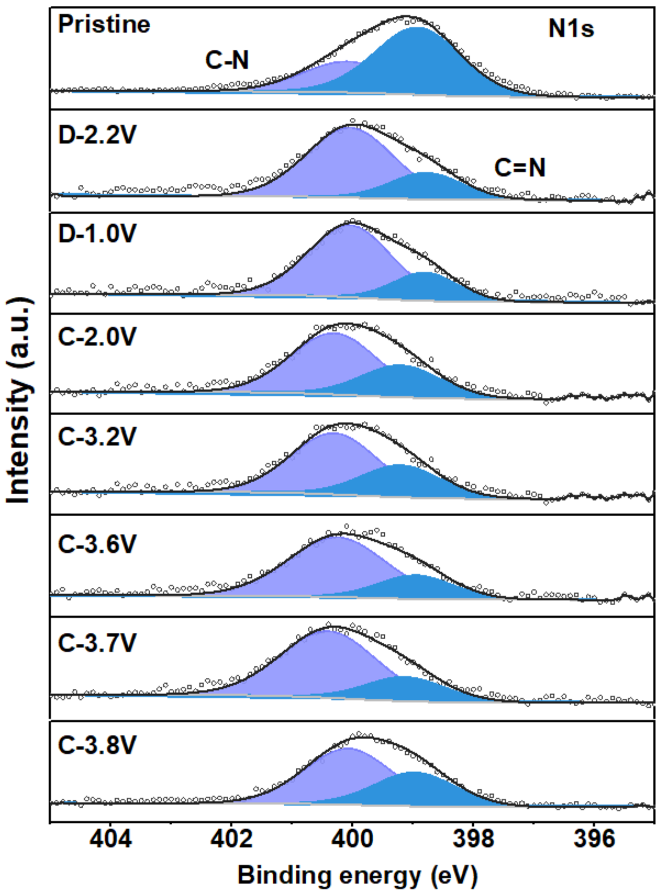

Figure S25. Ex-situ XPS N 1s spectra of the **2D-CuTABQ** electrode were recorded at different potentials during cycling.

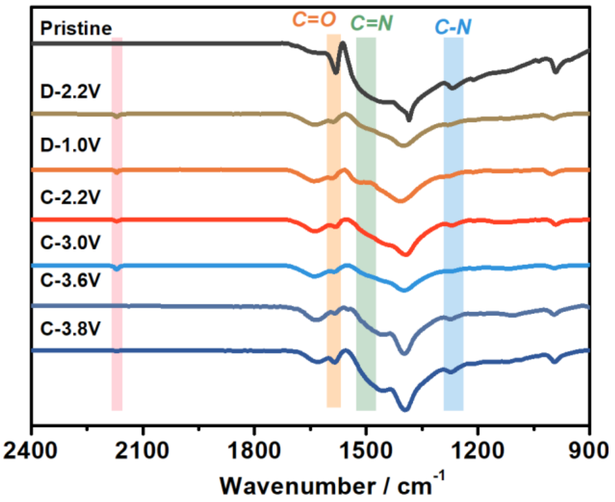

Figure S26. Ex-situ FTIR spectra of the **1D-CuTABQ** electrode were recorded at different potentials during cycling.

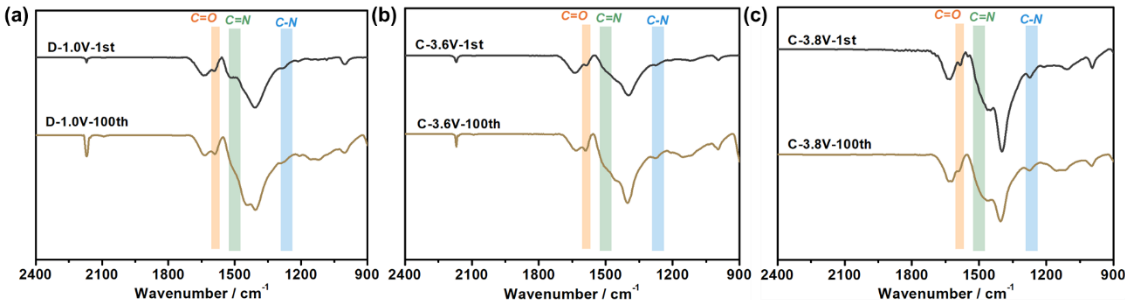

Figure S27. Ex situ FT-IR spectra of **1D-CuTABQ** electrode recorded after long-term cycling (100 cycles at 1 A g^{-1}).

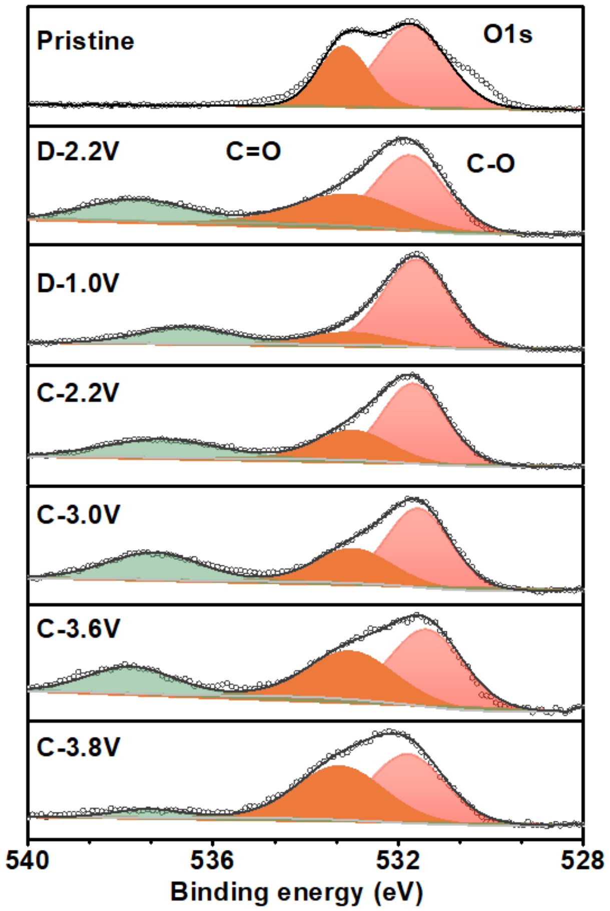

Figure S28. Ex situ O 1s XPS spectra of the **1D-CuTABQ** electrode were recorded at different potentials during cycling.

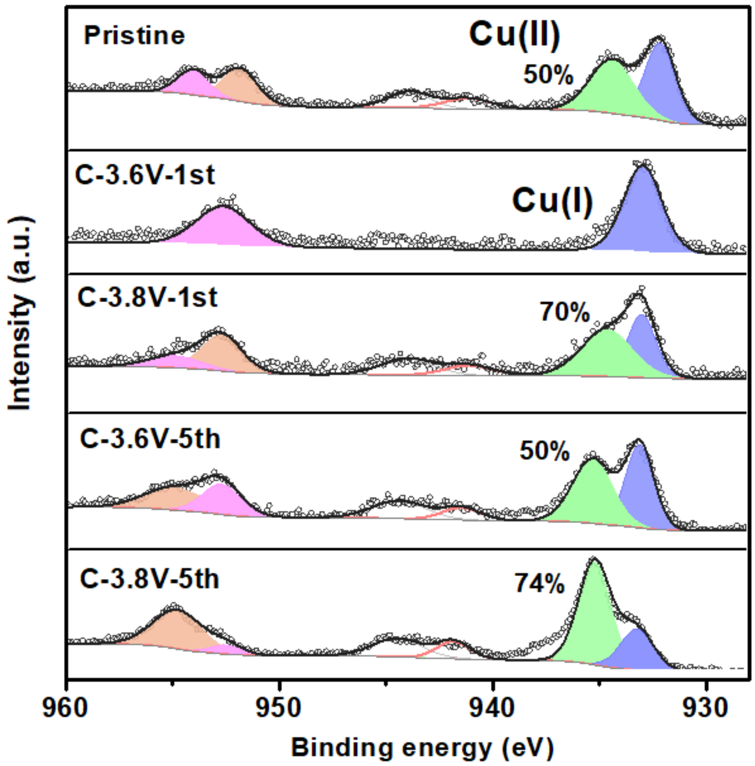

Figure 29. Ex situ Cu 2p XPS spectra of the **2D-CuTABQ** electrode were recorded at different potentials during different cycles.

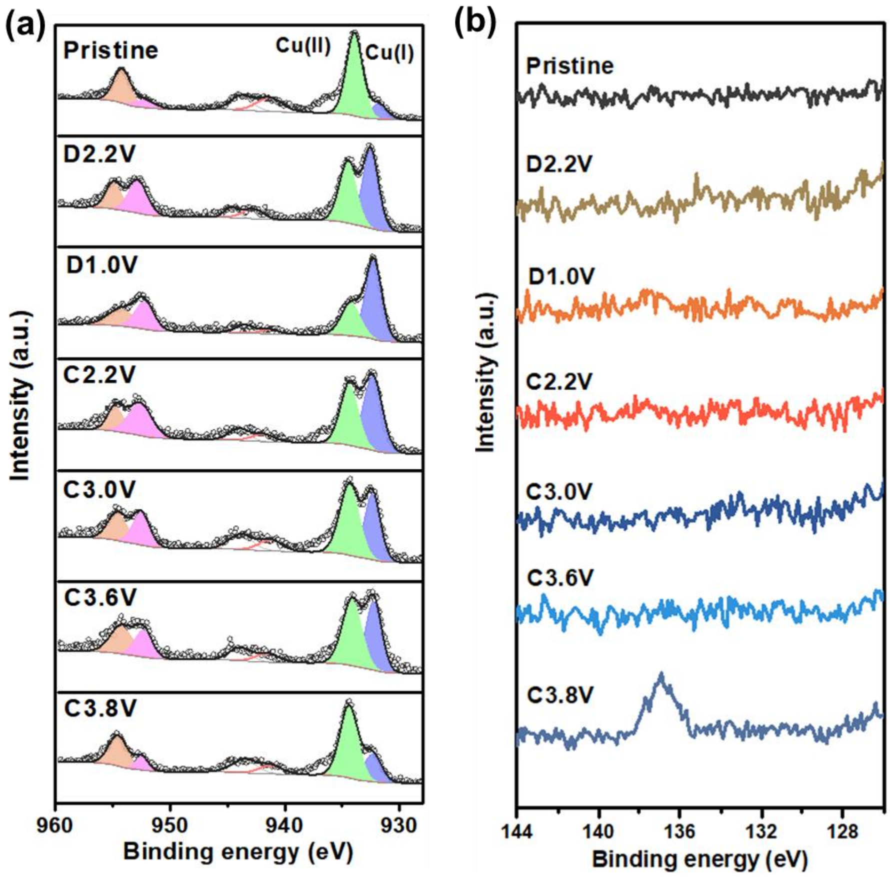

Figure S30. (a) Ex situ Cu 2p and (b) P 2p XPS spectra of the **1D-CuTABQ** electrode were recorded at different potentials during cycling.

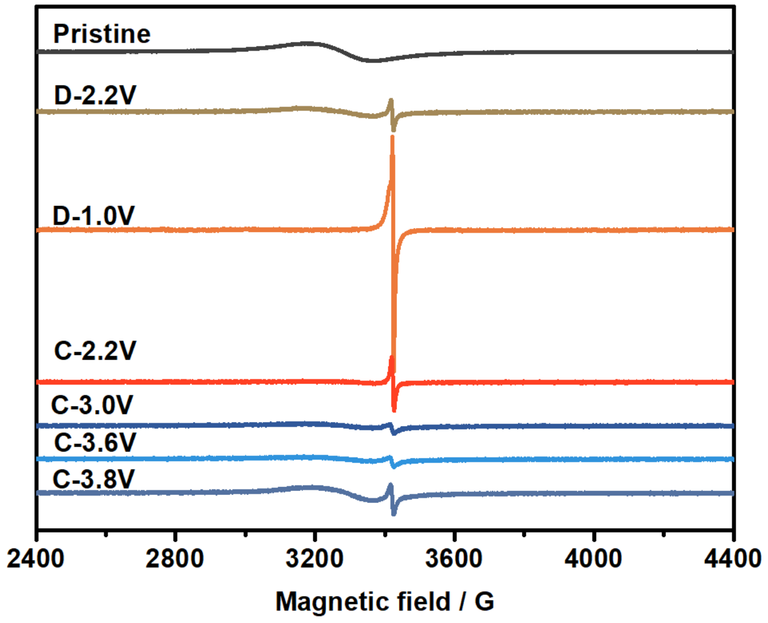

Figure S31. Ex-situ EPR spectra of the **1D-CuTABQ** electrode were recorded at different potentials.

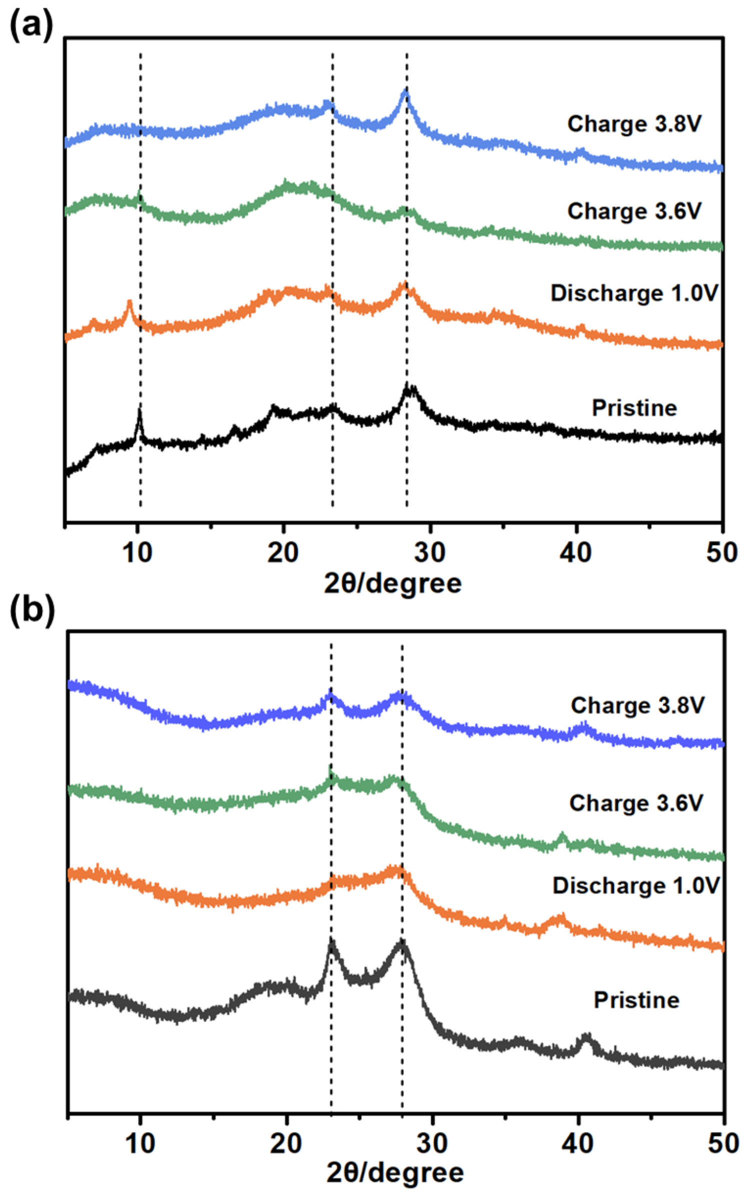

Figure S32. PXRD patterns of the (a) **2D-CuTABQ** and (b) **1D-CuTABQ** electrode during cycling.

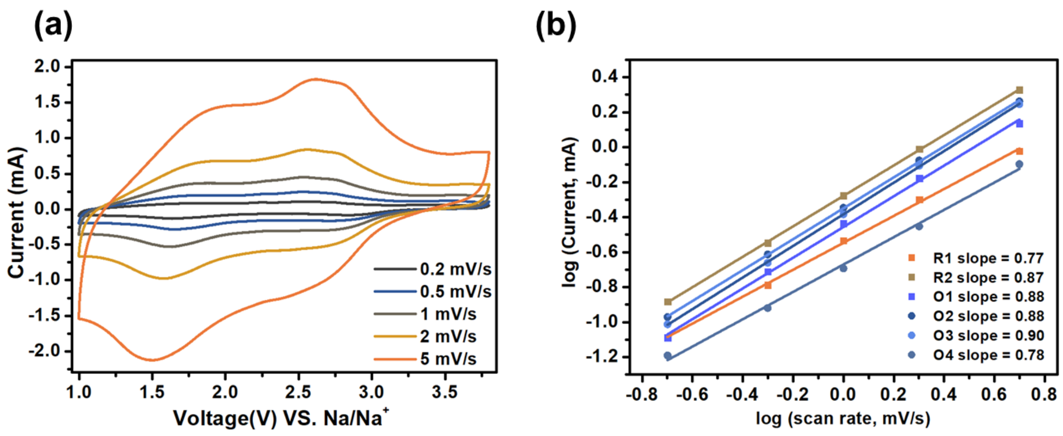

Figure S33. (a) CV curves at different sweeping rates from 0.1 to 5 mV/s for **2D-CuTABQ** within the voltage range of 1.0-3.8 V. (b) The corresponding plots of log(*i*) *vs.* log(*v*) for CV curves at different scan rates.

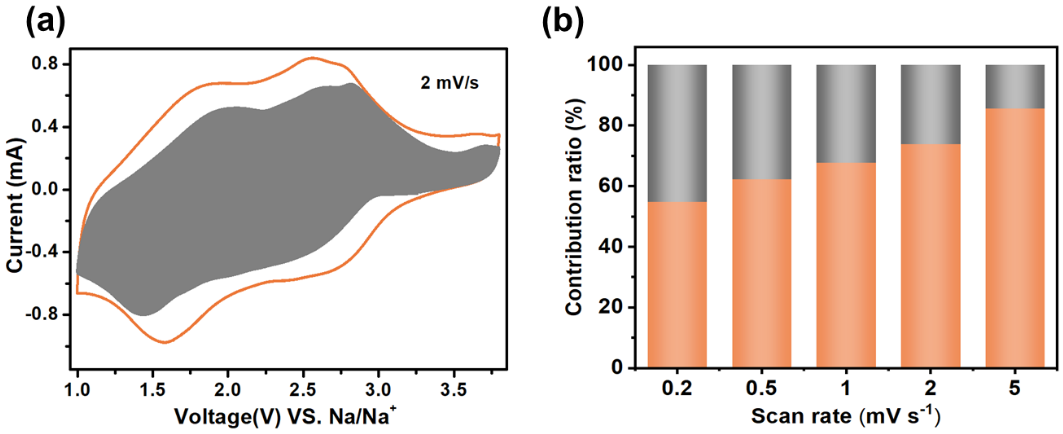

Figure S34. (a) Capacitive and diffusion currents contributed to the charge-storage of **2D-CuTABQ** electrodes at the rate of 2 mV s^{-1} within the voltage range of 1.0-3.8 V. (b) Contribution ratio of diffusion- and capacitive-controlled capacities at different scan rates for **2D-CuTABQ** electrodes. The capacitive contributions are calculated to be 55%, 62%, 68%, 74%, and 86% at the scan rates of 0.2, 0.5, 1.0, 2.0 and 5.0 mV s^{-1}, respectively.

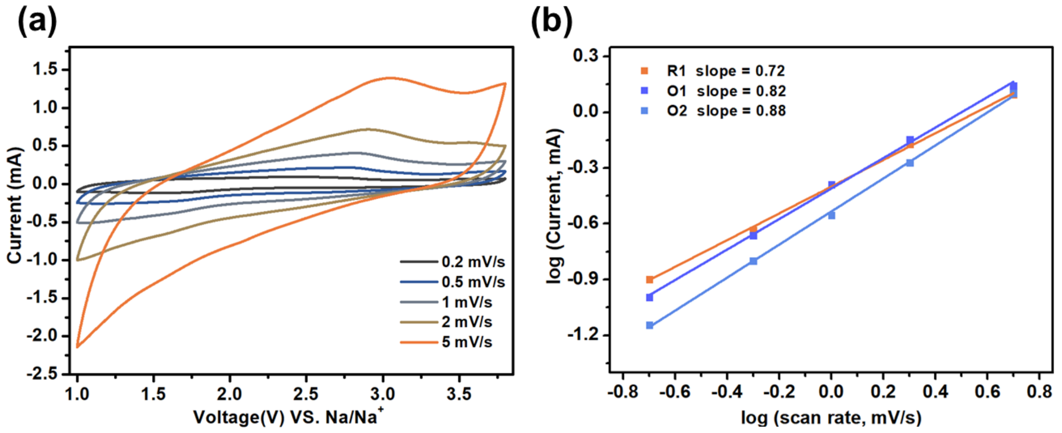

Figure S35. (a) CV curves at different sweeping rates from 0.1 to 5 mV/s for **1D-CuTABQ** within the voltage range of 1.0-3.8 V. (b) The corresponding plots of log(*i*) *vs.* log(*v*) for CV curves at different scan rates.

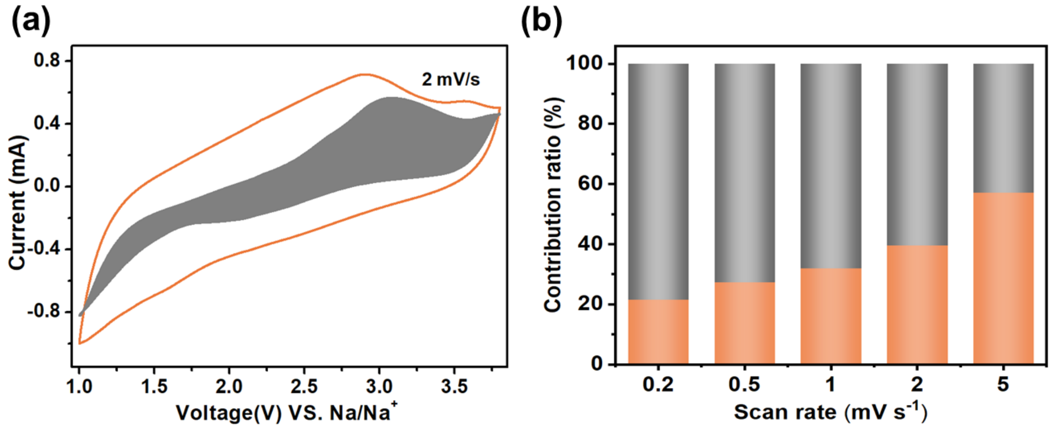

Figure S36. (a) Capacitive and diffusion currents contributed to the charge-storage of **1D-CuTABQ** electrodes at the rate of 2 mV s^{-1} within the voltage range of 1.0-3.8 V. (b) Contribution ratio of diffusion- and capacitive-controlled capacities at different scan rates for **1D-CuTABQ** electrodes. The capacitive contributions are calculated to be 21%, 27%, 32%, 39%, and 57% at the scan rates of 0.2, 0.5, 1.0, 2.0 and 5.0 mV s^{-1}, respectively.

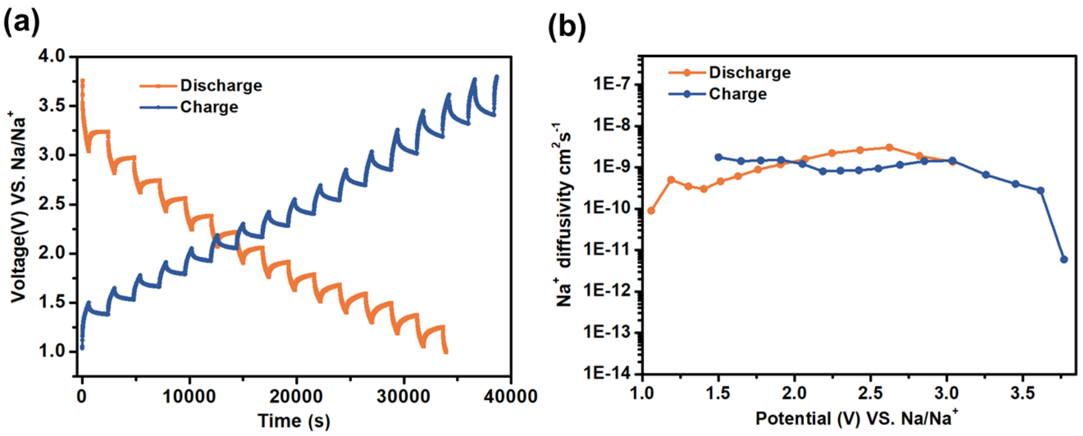

Figure S37. (a) GITT curves for **2D-CuTABQ** within the voltage range of 1.0-3.8 V. The experiment was tested at constant current pulse of 100 mA g^{-1} for 10 min followed by a relaxation period of 30 min. (d) Diffusivity calculated from GITT methods versus potential.

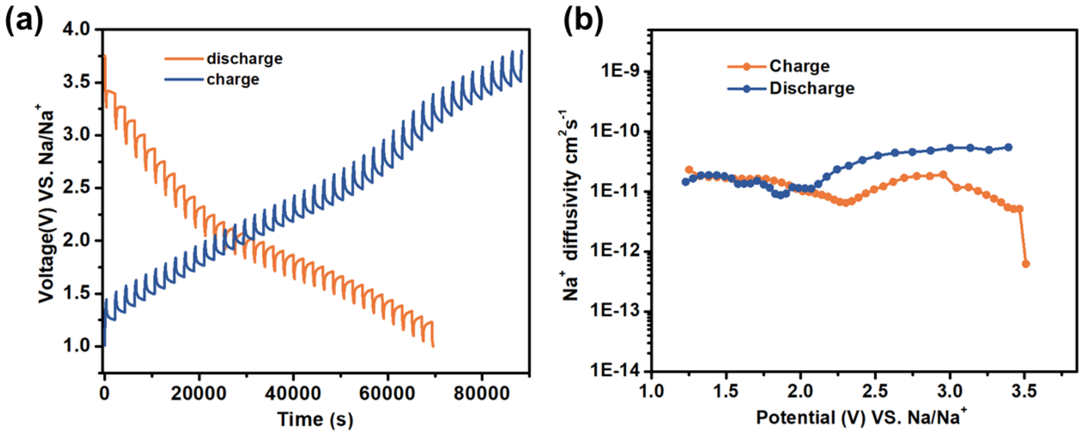

Figure S38. (a) GITT curves for **1D-CuTABQ** within the voltage range of 1.0-3.8 V. The experiment was tested at constant current pulse of 100 mA g^{-1} for 10 min followed by a relaxation period of 30 min. (d) Diffusivity calculated from GITT methods versus potential.

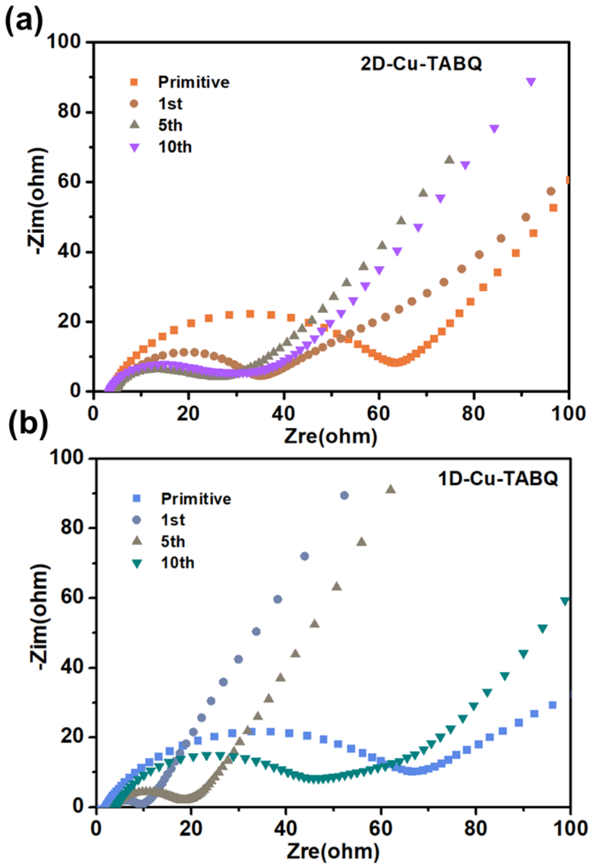

Figure S39. The Nyquist plots of the discharged (a) **2D-CuTABQ** and (b) **1D-CuTABQ** electrodes at different number of cycles within the voltage range of 1.0-3.8V.

References

[1] a) A. D. Becke, *J. Chem. Phys.*, 1993, **98**, 5648-5652; b) C. Lee, W. Yang and R. G. Parr, *Phys. Rev. B*, 1988, **37**, 785-789.

[2] a) W. J. Hehre, R. Ditchfield and J. A. Pople, *J. Chem. Phys.*, 1972, **56**, 2257-2261; b) M. M. Francl, W. J. Pietro, W. J. Hehre, J. S. Binkley, M. S. Gordon, D. J. DeFrees and J. A. Pople, *J. Chem. Phys.*, 1982, **77**, 3654-3665.

[3] Gaussian 16, Revision C.01, M. J. Frisch, G. W. Trucks, H. B. Schlegel, G. E. Scuseria, M. A. Robb, J. R. Cheeseman, G. Scalmani, V. Barone, G. A. Petersson, H. Nakatsuji, X. Li, M. Caricato, A. V. Marenich, J. Bloino, B. G. Janesko, R. Gomperts, B. Mennucci, H. P. Hratchian, J. V. Ortiz, A. F. Izmaylov, J. L. Sonnenberg, D. Williams-Young, F. Ding, F. Lipparini, F. Egidi, J. Goings, B. Peng, A. Petrone, T. Henderson, D. Ranasinghe, V. G. Zakrzewski, J. Gao, N. Rega, G. Zheng, W. Liang, M. Hada, M. Ehara, K. Toyota, R. Fukuda, J. Hasegawa, M. Ishida, T. Nakajima, Y. Honda, O. Kitao, H. Nakai, T. Vreven, K. Throssell, J. A. Montgomery, Jr., J. E. Peralta, F. Ogliaro, M. J. Bearpark, J. J. Heyd, E. N. Brothers, K. N. Kudin, V. N. Staroverov, T. A. Keith, R. Kobayashi, J. Normand, K. Raghavachari, A. P. Rendell, J. C. Burant, S. S. Iyengar, J. Tomasi, M. Cossi, J. M. Millam, M. Klene, C. Adamo, R. Cammi, J. W. Ochterski, R. L. Martin, K. Morokuma, O. Farkas, J. B. Foresman, and D. J. Fox, Gaussian, Inc., Wallingford CT, 2016.

[4] T. Lu and F. Chen, *J. Comput. Chem.*, 2012, **33**, 580-592.

[5] W. Humphrey, A. Dalke and K. Schulten, *J. Mol. Graph.*, 1996, **14**, 33-38.

[6] a) G. Kresse and J. Furthmüller, *Phys. Rev. B*, 1996, **54**, 11169-11186; b) P.E. Blochl, *Phys. Rev. B*, 1994, **50**, 17953-17979.

[7] J. P. Perdew, K. Burke and M. Ernzerhof, *Phys. Rev. Lett.*, 1996, **77**, 3865-3868.

[8] a) V. I. Anisimov, F. Aryasetiawan and A. I. Lichtenstein, *J. Phys.: Condens. Mat.*, 1997, **9**, 767-808. ; b) S. L. Dudarev, G. A. Botton, S. Y. Savrasov, C. J. Humphreys and A. P. Sutton, *Phys. Rev. B*, 1998, **57**, 1505-1509.

[9] S. Grimme, *J. Comput. Chem.*, 2006, **27**, 1787-1799.

[10] H. J. Monkhorst and J. D. Pack, *Phys. Rev. B*, 1976, **13**, 5188-5192.

[11] L. Liu, L. Li, M. E. Ziebel, T. D. Harris, *J. Am. Chem. Soc.* **2020**, *142*, 4705-4713.

[12] a) J. A. DeGayner, I. R. Jeon, L. Sun, M. Dincă, T. D. Harris, *J. Am. Chem. Soc.* **2017**, *139*, 4175-4184; b) R. Jeon Ie, B. Negru, R. P. Van Duyne, T. D. Harris, *J. Am. Chem. Soc.* **2015**, *137*, 15699-15702.

[13] G. Ferey, F. Millange, M. Morcrette, C. Serre, M.-L. Doublet, J.-M. Greneche, J.-M. Tarascon, *Angew.Chem.Int. Ed.* **2007**, *46*, 3259-3263.

[14] Z. Zhang, H. Yoshikawa, K. Awaga, *J. Am. Chem. Soc.* **2014**, *136*, 16112-16115.

[15] W. Kaveevivitchai, A. J. Jacobson, *J. Power Sources* **2015**, *278*, 265-273.

[16] Z. Peng, X. Yi, Z. Liu, J. Shang, D. Wang, *ACS Appl. Mater. Interfaces* **2016**, *8*, 14578-14585.

[17] Z. Zhang, H. Yoshikawa, K. Awaga, *Chem. Mater.* **2016**, *28*, 1298-1303.

[18] K. Taniguchi, J. Chen, Y. Sekine, H. Miyasaka, *Chem. Mater.* **2017**, *29*, 10053-10059.

[19] C. Li, C. Zhang, J. Xie, K. B. Wang, J. Z. Li, Q. C. Zhang, *Chem. Eng. J.* **2021**, *404*.

[20] D. Rambabu, A. E. Lakraychi, J. Wang, L. Sieuw, D. Gupta, P. Apostol, G. Chanteux, T. Goossens, K. Robeyns, A. Vlad, *J. Am. Chem. Soc.* **2021**, *143*, 11641-11650.

[21] K. Wada, K. Sakaushi, S. Sasaki, H. Nishihara, *Angew.Chem.Int. Ed.* **2018**, *57*, 8886-8890.

[22] H. Nagatomi, N. Yanai, T. Yamada, K. Shiraishi, N. Kimizuka, *Chem. Eur.J.* **2018**, *24*, 1806-1810.

[23] S. Gu, Z. Bai, S. Majumder, B. Huang, G. Chen, *J. Power Sources* **2019**, *429*, 22-29.

[24] R. R. Kapaev, S. Olthof, I. S. Zhidkov, E. Z. Kurmaev, K. J. Stevenson, K. Meerholz, P. A. Troshin, *Chem. Mater.* **2019**, *31*, 5197-5205.

[25] C.-H. Chang, A.-C. Li, I. Popovs, W. Kaveevivitchai, J.-L. Chen, K.-C. Chou, T.-S. Kuo, T.-H. Chen, *J. Mater. Chem. A* **2019**, *7*, 23770-23774.

[26] K. Wada, H. Maeda, T. Tsuji, K. Sakaushi, S. Sasaki, H. Nishihara, *Inorg. Chem.* **2020**, *59*, 10604-10610.

[27] Q. Jiang, P. Xiong, J. Liu, Z. Xie, Q. Wang, X. Q. Yang, E. Hu, Y. Cao, J. Sun, Y. Xu, L. Chen, *Angew. Chem. Int. Ed.* **2020**, *59*, 5273-5277.

[28] Z. Wang, G. Wang, H. Qi, M. Wang, M. Wang, S. Park, H. Wang, M. Yu, U. Kaiser, A. Fery, S. Zhou, R. Dong, X. Feng, *Chem. Sci.* **2020**, *11*, 7665-7671.

[29] M. E. Ziebel, C. A. Gaggioli, A. B. Turkiewicz, W. Ryu, L. Gagliardi, J. R. Long, *J. Am. Chem. Soc.* **2020**, *142*, 2653-2664.

[30] Z. Wu, D. Adekoya, X. Huang, M. J. Kiefel, J. Xie, W. Xu, Q. Zhang, D. Zhu, S. Zhang, *ACS Nano* **2020**, *14*, 12016-12026.

[31] M. L. Aubrey, J. R. Long, *J. Am. Chem. Soc.* **2015**, *137*, 13594-13602.

[32] C. Fang, Y. Huang, L. Yuan, Y. Liu, W. Chen, Y. Huang, K. Chen, J. Han, Q. Liu, Y. Huang, *Angew. Chem. Int. Ed.* **2017**, *56*, 6793-6797.

[33] H. J. Kim, Y. Kim, J. Shim, K. H. Jung, M. S. Jung, H. Kim, J. C. Lee, K. T. Lee,

ACS Appl. Mater. Interfaces **2018**, *10*, 3479-3486.
[34] Y. Wu, Y. Chen, M. Tang, S. Zhu, C. Jiang, S. Zhuo, C. Wang, *Chem. Commun.* **2019**, *55*, 10856-10859.
[35] Y. Chen, Q. Zhu, K. Fan, Y. Gu, M. Sun, Z. Li, C. Zhang, Y. Wu, Q. Wang, S. Xu, J. Ma, C. Wang, W. Hu, *Angew. Chem. Int. Ed.* **2021**, *60*, 18769-18776.
